# Neonatal endotoxin stimulation is associated with a long-term bronchiolar epithelial expression of innate immune and anti-allergic markers that attenuates the allergic response

**DOI:** 10.1371/journal.pone.0226233

**Published:** 2020-05-07

**Authors:** Luciana Noemi García, Carolina Leimgruber, Juan Pablo Nicola, Amado Alfredo Quintar, Cristina Alicia Maldonado

**Affiliations:** 1 Centro de Microscopía Electrónica, Universidad Nacional de Córdoba, Facultad de Ciencias Médicas, Córdoba, Argentina; 2 Consejo Nacional de Investigaciones Científicas y Técnicas (CONICET), Instituto de Investigaciones en Ciencias de la Salud (INICSA), Córdoba, Argentina; 3 Departamento de Bioquímica Clínica, Universidad Nacional de Córdoba, Facultad de Ciencias Químicas, Córdoba, Argentina; 4 Consejo Nacional de Investigaciones Científicas y Técnicas (CONICET), Centro de Investigaciones en Bioquímica Clínica e Inmunología (CIBICI), Córdoba, Argentina; University of Maryland School of Medicine, UNITED STATES

## Abstract

Allergic asthma is the most common phenotype of the pathology, having an early-onset in childhood and producing a Th2-driven airways remodeling process that leads to symptoms and pathophysiological changes. The avoidance of aeroallergen exposure in early life has been shown to prevent asthma, but without repeated success and with the underlying preventive mechanisms at the beginning of asthma far to be fully recognized. In the present study, we aimed to evaluate if neonatal LPS-induced boost in epithelial host defenses contribute to prevent OVA-induced asthma in adult mice. To this, we focused on the response of bronchiolar club cells (CC), which are highly specialized in maintaining the epithelial homeostasis in the lung. In these cells, neonatal LPS administration increased the expression of TLR4 and TNFα, as well as the immunodulatory/antiallergic proteins: club cell secretory protein (CCSP) and surfactant protein D (SP-D). LPS also prevented mucous metaplasia of club cells and reduced the epidermal growth factor receptor (EGFR)-dependent mucin overproduction, with mice displaying normal breathing patterns after OVA challenge. Furthermore, the overexpression of the epithelial Th2-related molecule TSLP was blunted, and normal TSLP and IL-4 levels were found in the bronchoalveolar lavage. A lower eosinophilia was detected in LPS-pretreated mice, along with an increase in phagocytes and regulatory cells (CD4+CD25+FOXP3+ and CD4+IL-10+), together with higher levels of IL-12 and TNFα. In conclusion, our study demonstrates stable asthma-preventive epithelial effects promoted by neonatal LPS stimulation, leading to the presence of regulatory cells in the lung. These anti-allergic dynamic mechanisms would be overlaid in the epithelium, favored by an adequate epidemiological environment, during the development of asthma.

## Introduction

Asthma is a heterogeneous disease with diverse underlying processes and many clinical expressions. The most common phenotype is allergic asthma, which has an early-onset in childhood and it is associated with a familiar history of allergic diseases. Histologically, it is characterized by chronic airway inflammation, with activated mast cells and an increased number of eosinophils, T cells, natural killer T cells, and CD4+ Th2 cells that release interleukin IL-4, IL-13, and IL-5. Additionally, IgE-secreting B cells are induced during the asthmatic process [1, 2]. In this phenotype, the continuous exposure to allergens produces several consequences in the structure and function of the airways, with the establishment of a remodeling process including mucus hypersecretion, smooth muscle hyperplasia, subepithelial fibrosis, blood vessel proliferation and the infiltration of inflammatory cells [3]. Although the avoidance of airborne allergen exposure in early life has been tested in randomized clinical trials, it has not been fully successful in preventing asthma development, suggesting that there are underlying mechanisms that have not yet been completely identified [4, 5].

The progressive rise in allergic diseases in recent decades suggests the involvement of environmental factors in their pathophysiology [6]. Based on epidemiological evidence, the hygiene hypothesis infers that the reduction of early life infections due to the modern lifestyle weakens protective effects against allergic disorders [7]. In agreement, multiple studies have revealed a low childhood prevalence of asthma in rural areas compared with urban areas, which was related to perinatal microbial exposure originating from the high levels of endotoxins present in dust samples [8–12]. Moreover, mouse models have identified potential immune mechanisms in which environmental microbial stimulation of the airways reduces allergic inflammation in the offspring, favoring homeostatic responses [13–22]. However, experimental studies also indicate that the level of lipopolysaccharide (LPS) exposure can determine the type of inflammatory response generated and provide a potential mechanistic explanation to epidemiological data on endotoxin exposure and asthma prevalence. Thus, the exposure to high-level LPS with allergic antigens results in increased antigen-specific Th 1 responses, whereas a low dose of LPS could promote Th2 sensitization, with LPS acting as a Th2 adjuvant [23–25]. In steady state conditions, the homeostasis of the airways relies on bronchioalveolar cells [26, 27]. For this purpose, airway epithelial cells (AECs) express inflammatory, anti-inflammatory, chemoattractants, antimicrobial mediators, and pattern recognition receptors to detect environmental molecules such as endotoxin and to initiate an innate immune response by activating dendritic cells [28]. This link between innate and adaptive immunity has revealed a significant role of AECs in lung immunity, and highlighted that an abnormal epithelial response may lead to a chronic inflammatory response [29]. When AECs come into contact with inhaled stimuli, which contain multiple proteolytic allergens, they are induced to produce ROS and pro-Th2 cytokines such as TSLP, IL-25, and IL-33 [30–32]. There is also increasing evidence concerning AECs intrinsic alterations in childhood asthma, which make the airways more vulnerable to airborne allergens and predispose them to Th2 responses [33–36]. These experimental data have indicated that AECs are essential controllers of the immune response to allergens, and may be an early player that biases a Th2 response in the immature immunity system. Therefore, AECs play a particular role since they are situated at the crossroads of the innate host defense and allergic inflammation.

Such contrasting epithelial activities are clearly exemplified by bronchiolar club cells. They are stem/progenitor cells of the AECs airways and perform a myriad of homeostatic mechanisms, including detoxification of xenobiotics [37, 38]. In addition, CC directly contribute to the host defenses by secreting monocyte and neutrophil chemoattractants, the antibacterial collectin SP-D and the immunomodulatory CCSP [39–45]. However, under allergic genetic predisposition, CC can also activate a Th2-inflammation via the IL-4 receptor, thereby driving eosinophil accumulation by producing eotaxin. Furthermore, they are the principal cells that undergo EGFR-mediated mucous metaplasia, as demonstrated in experimental models of asthma [32] [46–48]. Interestingly, both SP-D and CCSP play a direct role in suppressing allergic inflammation both *in vivo* and *in vitro*, leading to an increase in Th1 cytokines under LPS stimulation. Moreover, there are many investigations that have reported a reduction in these mediators in allergic/asthmatic patients, as well as in mouse models of asthma [39, 42, 43, 49–56].

The potential of CC to respond to a Th1 inflammatory stimulus by activating epithelial protective mechanisms has often been used in studies to determine whether this homeostatic role of the epithelium prevents the development of Th2 inflammation. In a previous study, we reported that in the adult mouse, the pre-exposition to LPS prior to allergen sensitization avoids mucous metaplasia of CC, and consequently, the loss of anti-allergic products. We also observed a reduction of eosinophil influx, IL-4 levels and airway hyperreactivity, whereas the Th1-related cytokines IL-12 and Interferon-gamma were enhanced [57]. Considering that early life represents a better window of opportunity for triggering an appropriate maturation of innate immunity, in the present study we evaluated whether the LPS stimulation during the neonatal period provides a better asthma-preventive effect by protecting adult AECs from Th2-driven inflammation.

## Materials and methods

### Animals

Balb/c mice were provided by Fun Vet (Universidad Nacional de La Plata, Argentina) and housed under controlled temperature and lighting conditions, with free access to tap water and commercial lab chow (GEPSA FEEDS, Buenos Aires, Argentina). Animals were randomly assigned to four groups (n = 6 each), and the experiments were repeated at least three times.

Animal care and experiments were conducted following the recommendations of the International Guiding Principles for Biomedical Research Involving Animals and approved by the Institutional Animal Care and Use Committee, CICUAL of the School of Medicine, National University of Córdoba, (Ref 97/20). During the airway challenge, animals were lightly anesthetized by inhaled isoflurane before the dropwise delivery of the volume into the nares using a pipetman; which allowed a quicker recovery time. For Bronchoalveolar lavage and lung collection, mice were anesthetized (i.p.) with a mixture of ketamine (60 mg/kg) and xylazine (15 mg/kg) and immediately sacrificed by exsanguination.

### Experimental design

#### Neonatal treatment

Balb/c mice offspring were exposed to intranasal applications on day 3 after birth and subsequently on every other day up to 13 days of life. While one group of animals were treated with PBS, the other received 1μg/5μl of LPS (*Escherichia coli* O55:B5 Sigma-Aldrich; St. Louis, MO, USA) according to protocols optimized for volume [15], treatment timing [19], and according to evidence indicating that this dose induces antigen-specific Th1 responses and previous studies [17, 23].

#### Allergen sensitization in weaned animals

At the age of 4 weeks, animals were sensitized by subsequent i.p injections of 0.1 ml of Ovalbumin (OVA) grade VI (100μg/100 μl, Sigma-Aldrich) absorbed to 1 mg of inject Alum (Pierce Rockford, USA) on the 4^th^ and 6^th^ weeks of life.

#### Airway challenge in puberty and adulthood

**Fig 1:**
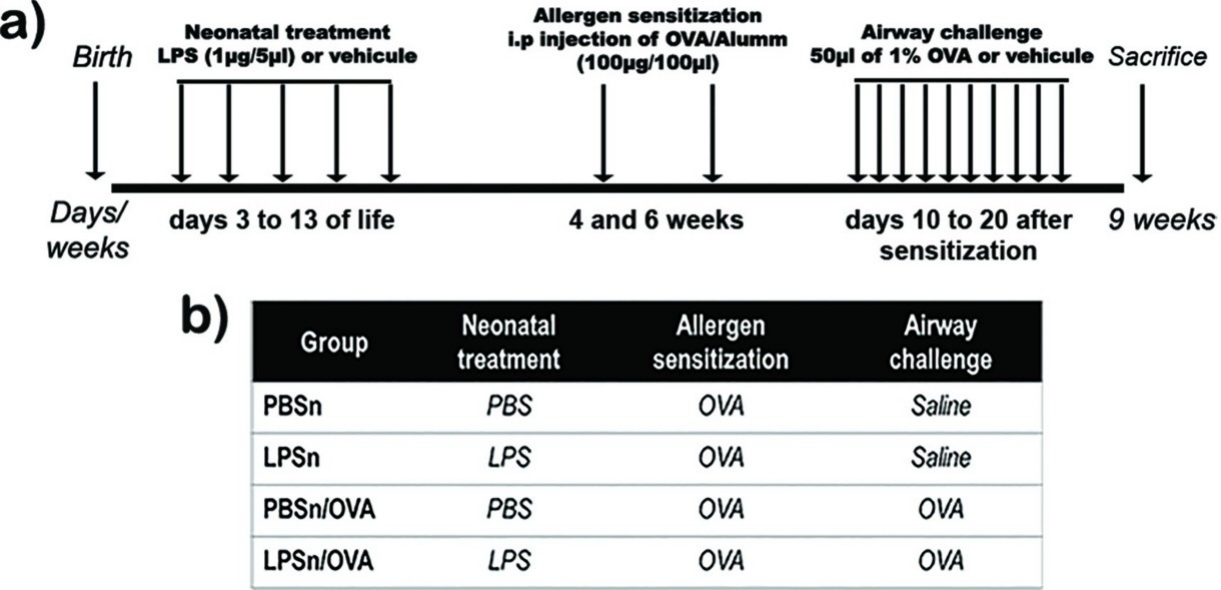
Experimental design. (A) Timeline diagram and (B) table with protocols employed in this study. All data are representative of at least 12 animals per group in 3 independent experiments.

Ten days later (7h weeks of life), neonatally LPS-treated mice as well as PBS-exposed ones were divided into 2 groups. Whereas the LPSn/OVA and PBSn/OVA mice were challenged daily (on 10 consecutive days) by an intranasal application of 50μl of 1% OVA, the LPSn and PBSn mice were submitted to an intranasal application of saline (Fig 1). Then, after 24h, at 9h weeks of life, mice were sacrificed and processed according to the specific methods outlined below.

http://dx.doi.org/10.17504/protocols.io.bdwwi7fe

### Lung histopathology

A morphological analysis was performed in the right lungs of 3 mice per group, as previously described [54]. Briefly, in at least in 3 experiments, lungs were differentially fixed for either ultrastructural or histopathology analysis by intratracheal perfusion, and prepared for examination under a transmission electron microscope (Zeiss LEO 906E) or light microscope (Axiostar Plus, Zeiss, Germany).

### Mucous cell staining

The Alcian blue-periodic acid Schiff (AB-PAS) staining technique was used, as previously described [54], to identify mucous-secreting cells in the bronchiolar epithelium. Photomicrographs at x400 were taken using a light microscope equipped with a digital camera (Axiocam ERc5s). A total of 20–30 bronchioles (900–1700 μm diameter) per mouse were analyzed, and the number of epithelial AB-PAS positive cells per 100μm of basement membrane was quantified using Image J (NIH version 1.43).

### Immunohistochemical analysis of lung tissue

Immunohistochemical staining was performed as described elsewhere [57]. Briefly, after being blocked, the sections were incubated overnight at 4ºC with antibodies recognizing SP-D (1:1000-Chemicon, Temecula, CA, USA), TNFα (1:50—Hycult, Plymouth Meeting, USA), CCSP (Clara cell CC10 antibody 1:1000—Santa Cruz Biotechnology, Santa Cruz, CA, USA), Toll-like receptor (TLR) 4 (1:100- Santa Cruz Biotechnology), TSLP (1:200- Gene Tex, USA) or phosphorylated EGFR (pEGFR) (1:50—Santa Cruz Biotechnology), with the bound antibodies being detected using anti-rabbit (for SP-D, TNFα, TSLP and CCSP) or anti-goat (for TLR4 and pEGFR) biotin-labeled antibodies (Vector Laboratories, Burlingame, CA, USA) in 1% PBS-BSA. The sections were then incubated with ABC complex (VECTASTAIN Vector Labs, Southfield, MI, USA). Diaminobenzidinde (DAB, Sigma-Aldrich) was used as a chromogen substrate. The scoring of TSLP reactivity was performed with a computer-assisted imaging system (Image J by NIH, Bethesda, MD, USA), based on the intensity and the stained area measured in each field of vision, and expressed as epithelial pixels per total area.

http://dx.doi.org/10.17504/protocols.io.bdw9i7h6

### Bronchoalveolar lavage collection and cell counting

Bronchoalveolar lavage (BAL) was obtained (n = 9 mice/group in three different experiments) as described elsewhere [54]. Briefly, after three serial intra-tracheal instillations of 1 ml PBS, the cells obtained were centrifuged at 200g, resuspended and counted, with the supernatant being stored at -70ºC for ELISA.

For cytospin preparations, about 12.5x10^4^ cells from the pellets were cytocentrifuged onto slides, some of which were preserved at -70°C for immunofluorescence, while others were stained with May Grünwald-Giemsa (Biopur Diagnostic, Rosario, Argentina), after which the cells were counted and total count informed per BAL. These cell populations were evaluated for two samples per mouse, with a total of 2400 cells per group being counted.

### Immunofluorescence

Cytospin preparations (3 per mice) obtained from the BAL (3 mice per group) were withdrawn at room temperature and immediately fixed with 4% formaldehyde, permeabilized with 0.25% Triton X-100 in PBS and incubated for 1 h in 5% PBS-BSA to block non-specific binding. The slides were first double immunostained by incubating overnight at 4ºC with a mix of anti-CD4 conjugated with PerCP (BioLegend, San Diego, CA, USA) and anti IL-10 conjugated with PE (BD Biosciences Pharmingen, San Diego, CA), and then mounted using fluoromount containing 4'-6-diamidino-2-phenylindole (DAPI). Afterwards, the cells were viewed with Fluoview 1000 Confocal and laser scanning microscope, (Olympus, Tokyo, Japan), with serial x 60 microphotographs (10 per coverslide) being obtained and all double immunostained cells being evaluated for three different experiments in order to determine the relative percentages.

http://dx.doi.org/10.17504/protocols.io.bdxzi7p6

### Flow cytometry

Pellet cells obtained from BAL (n = 5 mice/group in three different experiments) were incubated for 30 min at 4°C with a mix of conjugated antibodies (Biolegend) for the following T-cell subset superficial markers: APC-Cy7 anti-mouse CD45 (1:600), FITC anti-mouse CD4 (1:200) and PerCP anti-mouse CD25 (1:200). Next, the cells were fixed (CITOFIX; BD Biosciences Pharmingen, San Diego, CA) for 20 min at 4°C and permeabilized with Perm/Wash (BD Biosciences Pharmigen), before being incubated with a dilution 1:30 of the APC anti-mouse forkhead box P3 (FOXP3) intracytoplasmic antibody (eBioscience) for 30 min at 4°C. Finally, the cells were washed, suspended in filtered PBS and analyzed by flow cytometry (1x105 events/experimental treatment), FACSCanto II Flow Cytometer, BD Biosciences, San Diego, CA, USA). Data analysis was carried out using the FlowJo software (Tree Star, Ashland, OR).

http://dx.doi.org/10.17504/protocols.io.bdx3i7qn

### Immunobloting

SP-D, TLR4, and TNFα levels were determined in total lung homogenates from 3 animals per group for three different experiments by western blot as previously described [54]. The total protein concentration was measured with a Bio-Rad kit (Bio-Rad Laboratories, Hercules, CA, USA) and 75μg/lane of adjusted denatured protein samples were separated on 12% SDS-PAGE and blotted onto a Hybond-C membrane (Amersham Pharmacia-GE, Piscataway, NJ, USA). Membranes were then blocked with 5% defatted dry milk in TBS/0.1% Tween 20, and incubated for 3h with one of the following antibodies: rabbit anti-SP-D (1:1000—Chemicon, rabbit anti-TNFα (1:50 –Hycult) or mouse anti-TLR4 (1:300 Abcam, Maryland, USA). Blots were incubated with a peroxidase-conjugated (HRP) anti-rabbit (Jackson Immunoresearch Labs Inc, West Grove, PA, USA), or anti-mouse (Jackson Immunoresearch) secondary antibodies at a 1:2000 dilution. Finally, the membranes were rinsed in TBS/0.1% Tween-20 and exposed to Pierce^™^ ECL Western Blotting Substrate (Thermo Fischer Scientific), following the manufacturer’s instructions. Emitted light was captured on Hyperfilm (Amersham-Pharmacia), and a densitometry analysis was performed by applying the Scion Image software (V. beta 4.0.2, Scion Image Corp., Frederick, MD, USA). In addition, β-actin expression was used as an internal control to confirm equivalent total protein loading using a mouse antibody (ACTB, 1:4000; mouse anti-mouse βactin; Sigma-Aldrich).

### Dot blot analysis

The CCSP protein expression was evaluated in lung homogenates using a Bio-Rad kit. Samples were then adjusted to 5μg/μl in PBS, pH 7.4, and 10μl of each sample were spotted onto a Hybond C membrane (Amersham Pharmacia). The membrane was blocked with 5% fat-free milk in PBS buffer for 1h, before being incubated for 3h with the rabbit primary antibody anti-CC10 1:500 (Santa Cruz Biotechnology) in blocking buffer at room temperature. After washing with TBS–Tween-20 buffer, the membrane was treated with a HRP-conjugated anti-rabbit antibody (Jackson Immunoresearch) and the next handle was as described above for Western blot. http://dx.doi.org/10.17504/protocols.io.bbyhipt6

### Cytokine detection by ELISA

Cytokine production was measured in the BAL supernatant, following the manufacturer´s instructions, by applying commercially available sandwich ELISA kits for IL-4 (BD Biosciences), IL-12 and TSLP (Biolegend, San Diego, CA, USA), as well as for TNFα and IFNγ (eBioscience, San Diego, CA, USA).

### RNA isolation and gene expression analysis

Total RNA was extracted from right lung tissue samples (~0,01mg) with Trizol reagent. RNA was subsequently purified using the Direct-zol RNA min prep kit (Zymo Research), following the manufacturer’s instructions, and quantified with a ND-1,000, NanoDrop spectrophotometer (Thermo Scientific) at 260 nm, 1μg of RNA was used as the template for reverse transcription, following the manufacturer’s instructions (EpiScript^TM^ Reverse Transcriptase System kit, Epicentre, USA), using random hexamer primers (Fermentas, Thermo Fisher Scientific, MA, USA) and a My Cicle rTM BIO-RAD (Thermal Cycler System, CA, USA).

Real-Time PCR analysis was performed on an ABI Prism 7500 detection system (Applied Biosystem, CA, USA) using Power SYBR Green PCR Master Mix (Applied Biosystems, Thermo Fisher Scientific). Relative changes in gene expression were calculated by the 2-ΔΔCt method normalized against the housekeeping gene 18S. The amplification efficiency for each pair of primers was calculated using standard curves generated by serial dilutions of cDNA, with all primers used being from Invitrogen (Buenos Aires, Argentina) and detailed below:

TSLPfp: 5’-AGAGAAATGACGGTACTCAGG-3’, TSLPrp: 5’-TTCTGGAGATTGCATGAAGGA-3’; 18sfp 5’-ATGCGGCGGCGTTATTCC-3’, 18srp: 5’-GCTATCAATCTGTCAATCCTGTCC-3’; CCSPfp: 5’-GATCGCCATCACAATCACTG-3’, CCSPrp: 5’-CTCTTGTGGGAGGGTATCCA-3’; SP-Dfp: 5’-TGGACCCAAAGGAGAGAATG-3’, SP-Drp: 5’-CATGCCAGGAGCACCTACTT-3’.

http://dx.doi.org/10.17504/protocols.io.bdx5i7q6

### Clinical score assessment of the degree of respiratory distress

For the three different protocols at days 7–10 of the allergen challenge, the breathing patterns of mice (n = 6/group) were recorded on video for the first minute after OVA instillation (S2 File and http://dx.doi.org/10.17504/protocols.io.bbymipu6). The values assigned to increasing signs of respiratory distress were adapted from the respiratory failure clinical score system developed by Wood [58]. This scoring was performed, via a double-blind procedure, by three different physician operators and analyzed by a non-parametric statistical test (see below).

### Statistical analysis

In general, the data obtained were analyzed by a one-way ANOVA, followed by a post-hoc comparison with the Tukey-Kramer test. In particular, for the analysis of clinical score data, we applied the Kruskal Wallis test. For all tests, a p<0.05 significance level was used. Statistical tests were performed using the InfoStat (Faculty of Agricultural Sciences, National University of Córdoba, Córdoba, Argentina) statistical program.

## Results

### Neonatal LPS stimulation prevented OVA-induced allergic airway inflammation triggered in puberty and adulthood

AECs act as a physical and biochemical barrier with an involvement in every stage of the inflammatory reaction. As a part of the epithelial-mesenchymal trophic unit, they have been suggested as a potential mechanism for the exaggerated response seen in asthma.[33]. Therefore, we initiated evaluating the effect of an early exposition to LPS (LPSn/OVA group) on an ulterior OVA allergic response in the airways, by analyzing the levels of cytokines and cellular inflammatory content in BAL as well as the breathing patterns recorded in the different mouse groups.

**Fig 2:**
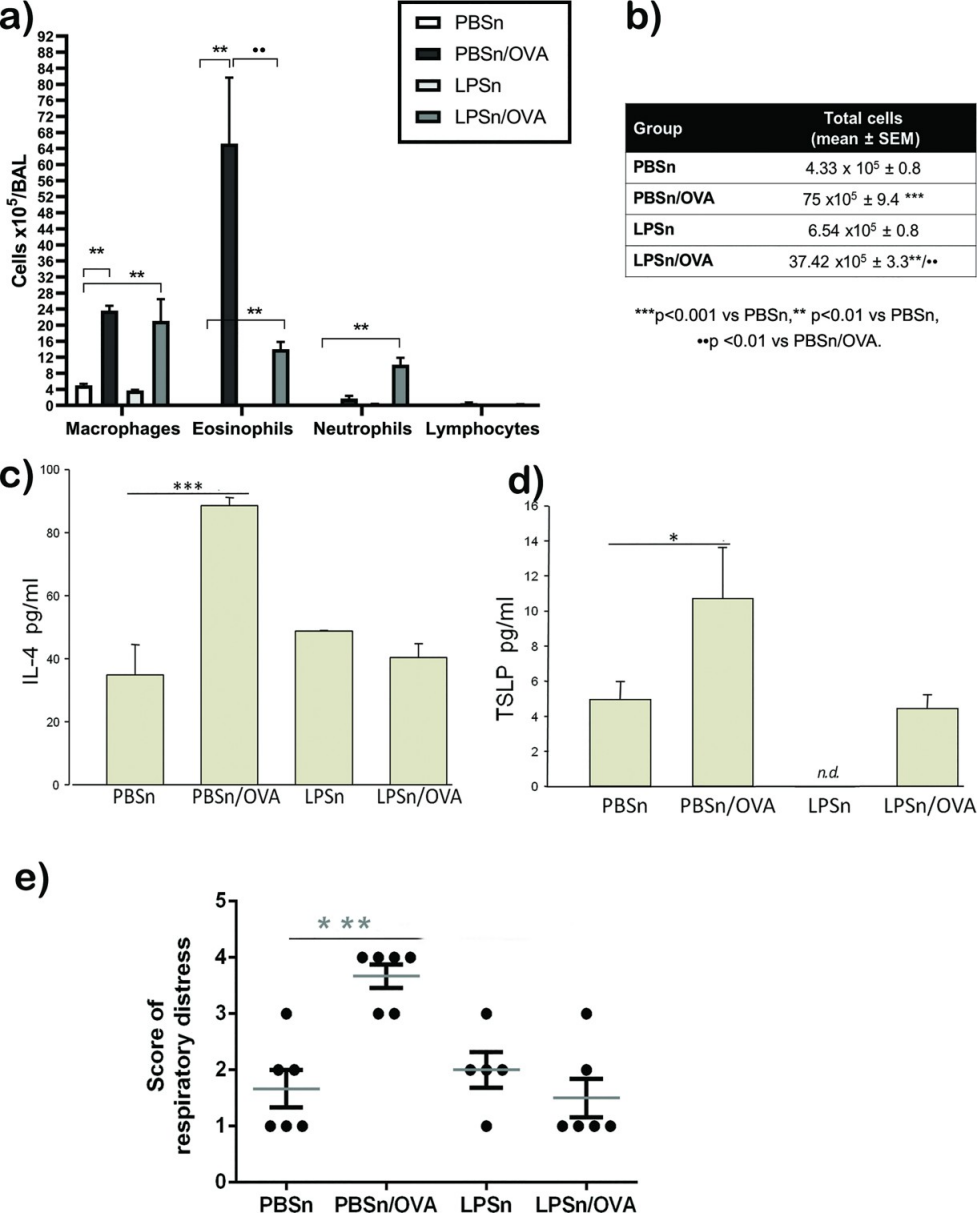
Allergic inflammatory state. (A) Differential quantification of cell populations in bronchoalveolar lavage (BAL). Bar graph representing the total number of macrophages, esosinophils, neutrophils and lymphocytes in BAL. (B) total cell count in BAL. (C) and (D): Levels of IL-4 and TSLP by ELISA. (E) Score of respiratory distress, representing increasing signs of distress obtained in the first minute after intranasal challenge in all groups. Data represent mean ± SD ***p<0.001 vs PBSn,** p<0.01 vs PBSn, *p<0.05 vs PBSn, ●● p <0.01 vs PBSn/OVA. All data are representative of at least 6 animals per group in 3 independent experiments.

Neonatal LPS treatment affected the development of experimental asthma triggered after weaning. As shown in Fig 2, the establishment of experimental asthma in 7–9 week old mice was largely prevented, as indicated by the significantly lower influx of both total inflammatory cells and eosinophils into the airway lumen of LPSn/OVA mice compared to the PBSn/OVA group (Fig 2A and 2B). In addition, the number of macrophages remained unchanged whereas neutrophils increased significantly (Fig 2A). Surprisingly, IL-4 and TSLP, both associated with Th2 inflammation, exhibited normal levels in the BAL of LPS pre-treated mice in spite of the allergen-challenge, while they were significantly higher in PBSn/OVA group (Fig 2C and 2D respectively). As expected, in the LPSn group, neither the BAL cell count nor IL-4 content were different from controls (Fig 2B and 2C). However, TSLP was remarkably reduced to non-detectable levels (Fig 2D).

To test whether the inflammatory parameters were accompanied by changes in the degree of respiratory distress, a clinical scoring system was applied (S2 File and S3 File). While most of the neonatal PBS-exposed mice displayed more signs of respiratory distress after an OVA challenge, the breathing pattern of LPSn/OVA mice was similar to that of control mice (Fig 2E).

### Neonatal LPS application promoted TNFα, IL-12, and IFNγ secretion as well as Treg cells in the airways microenvironment in front of OVA stimulation

**Fig 3:**
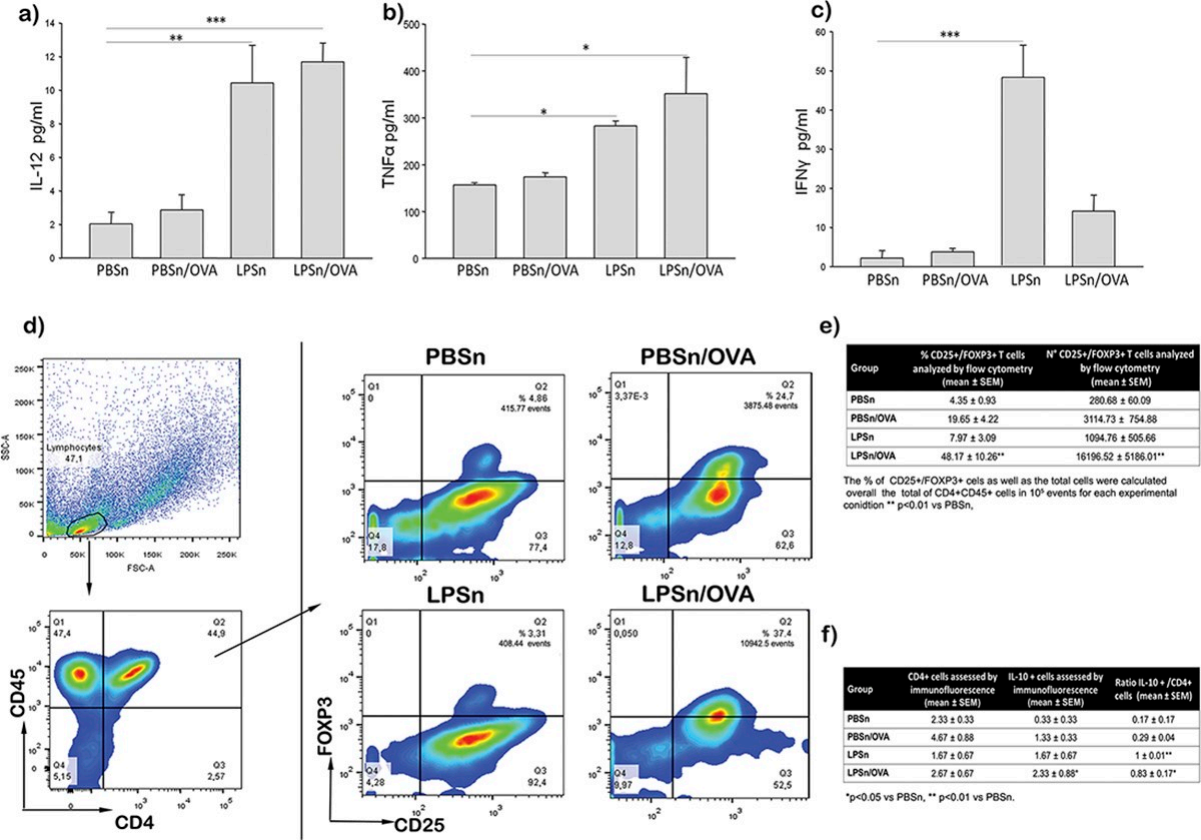
Modulatory response of the airway environment in BAL. (A), (B) and (C) IL-12, TNFα and IFNγ levels in bronchoalveolar lavage (BAL) by ELISA, respectively. (D) Representative experimental analysis by flow cytometry, of the percentage of CD25+FOXP3+ cells is shown in the Q2 quadrant. (E) Percentage of CD4+CD25+FOXP3+ cells and absolute number obtained for all groups by flow cytometry in BAL. (F) Immunofluorescence count of CD4+ and IL 10+, and the ratio of IL10+/CD4+cells, performed in cytospin. Data are represented as mean ±SEM, *p< 0.05 vs PBSn, **p<0.01 vs PBSn, ***p<0.001vs PBSn. All data are representative of at least 5 animals per group in 3 independent experiment.

After demonstrating the abrogation of a Th2 inflammatory response, we investigated whether neonatal LPS exposure influences the content of other components of the immune response in the BAL. In the milieu within the airway, LPS stimulus increased inflammatory cytokines such as IL-12 and TNFα in both the LPSn and LPSn/OVA mice groups (Fig 3A and 3B, respectively). In addition, it was noteworthy that LPS induced high levels of IFNγ (a prototypical Th1 cytokine) in the LPSn group, but not in LPSn/OVA animals (Fig 3C).

With this in mind, we further investigated the participation of regulatory T cells in this modulatory response by flow cytometry and the presence of IL-10 cells by immunofluorescence in BAL. We performed a flow cytometry analysis (Fig 3D) gating on the subset of CD4+/CD45+/CD25+/FOXP3+ Tregs. The percentage as well as the absolute number of CD25+/FOXP3+ Treg cells (Fig 3E) exhibited a higher influx after allergen challenge in the LPS-preexposed group (48.16% ± 10.2 LPSn/OVA vs 19.65% ± 4.22 PBSn/OVA—p<0,01- and 16296,52 ± 5185,01 cells vs 280,68 ± 60,09 cells—p<0,01). Meanwhile neither the percentage or the absolute number in the PBSnOVA group reached a significant change (p = 0.0510 and p = 0.6062 respectively). In addition, immunofluorescence performed on cytospins by confocal microscopy revealed an increased level of IL-10 positive cells in the LPSn/OVA group compared with PBSn animals (Fig 3F). Appreciably in the LPSn and LPSnOVA groups, almost every CD4+ cell also expressed IL10. We then calculated the IL-10+/CD4+ cell ratio that revealed a significant polarization of CD4 cells in both groups (LPSn and LPSn/OVA) compared to control (Fig 3F).

### Neonatal LPS exposure abrogated the development of mucous metaplasia and pro-allergic mediators in the bronchiolar epithelium

**Fig 4:**
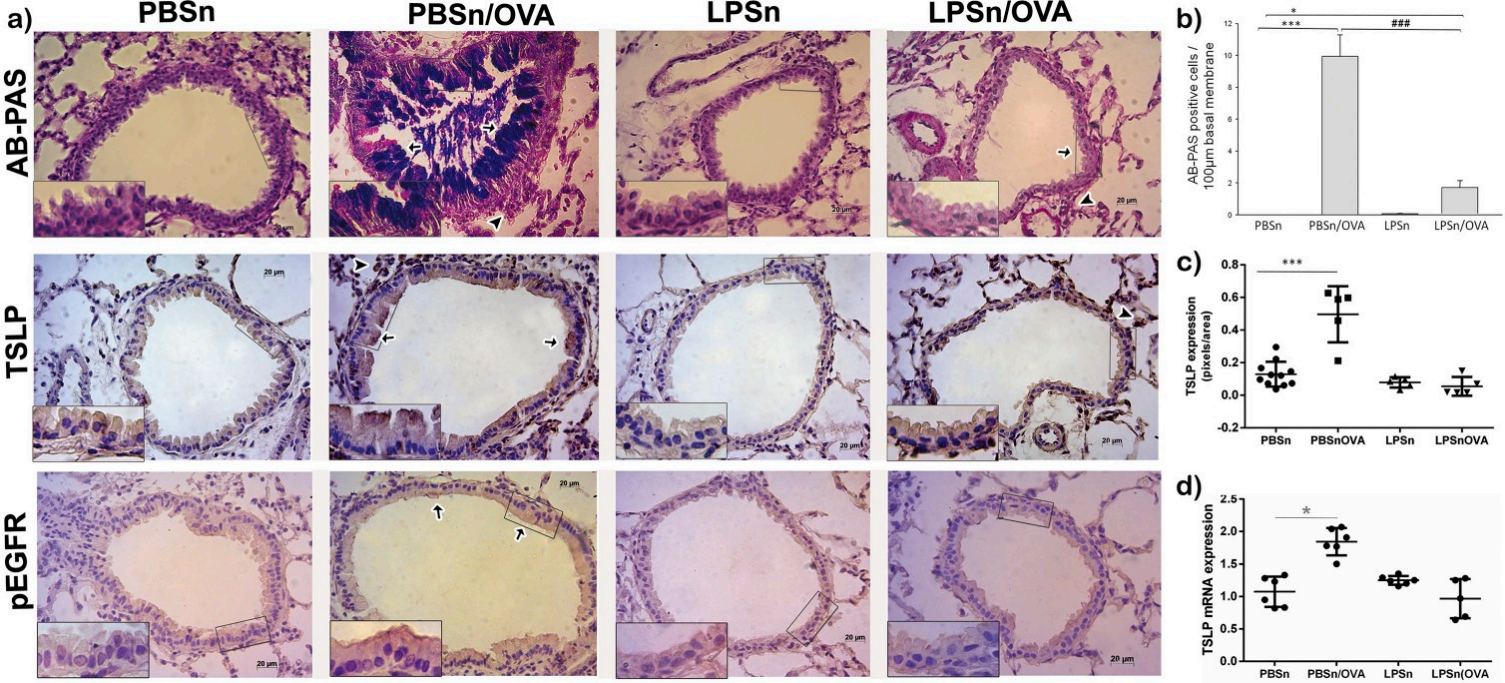
Mucous metaplasia analysis in club cells and epithelial TSLP expression. (A) Representative photomicrographs of Alcian blue-periodic acid Schiff (AB-PAS), TSLP and pEGFR staining of bronchiolar sections. Scale bars: 20μm. In AB-PAS panel, arrows indicate AB-PAS positive cells in the PBSn/OVA and LPSn/OVA groups, while arrowheads indicate infiltrating inflammatory cells. In the TSLP panel, arrows indicate positive cells in PBSn/OVA, although some cells (arrowhead) from the inflammatory response also expressed TSLP. The inset selection demonstrates the lack of staining in club cells of the LPSn/OVA groups. In pEGFR, panel arrows indicate positive cells in PBSn/OVA, while the inset demonstrates the apical expression of the activated receptor in CC. (B) Graph represents AB-PAS cell count per 100μm. (C) Graph represents morphometric analysis of TSLP staining. (D) TSLP mRNA expression by Real-Time PCR analysis. Graph represents fold increase in expression in lung tissue homogenate. Data are represented as mean ±SEM, *p<0.05 vs PBSn, ***p<0.001 vs PBSn, ### p<0.001 vs PBSn/OVA. All data are representative of at least 6 animals per group in 3 independent experiment.

We first analyzed changes in the expression of the specific epithelial molecules involved in the allergic process that are known to increase in the bronchiolar epithelium during asthma. As shown [57], OVA-allergic inflammation incited mucous cell metaplasia in the bronchiolar CC via EGFR signalling. Fig 4 shows an increased number of mucous secreting cells (AB-PAS panel in Fig 4A and Fig 4B) as well as the overexpression of phosphorylated-EGFR in the apical cytoplasm of CC in PBSn/OVA mice. In contrast, in LPSn/OVA mice, both pEGFR overexpression (pEGFR panel in Fig 4A) and the mucous metaplasia (Fig 4B) were largely reduced by the neonatal endotoxin-treatment.

We also studied the effects of neonatal LPS on the expression of TSLP, an epithelial cell cytokine that promotes Th2 differentiation after allergen contact. Similarly to pEGFR and mucous metaplasia induction, bronchiolar epithelial cells of PBSn/OVA group showed a strong TSLP immunoreactivity in the apical cytoplasm, while CC of LPSn/OVA animals did not result in overexpression of TSLP (TSLP panel in Fig 4A and 4C). These results were corroborated by qPCR (Fig 4D), which showed that lung TSLP mRNA almost duplicated its expression in PBSn/OVA animals (1.85 ± 0.09 PBSn/OVA vs 1 ± 0.1 PBSn), while remaining unchanged in LPSn/OVA mice (1.12 ± 0.19).

**Fig 5:**
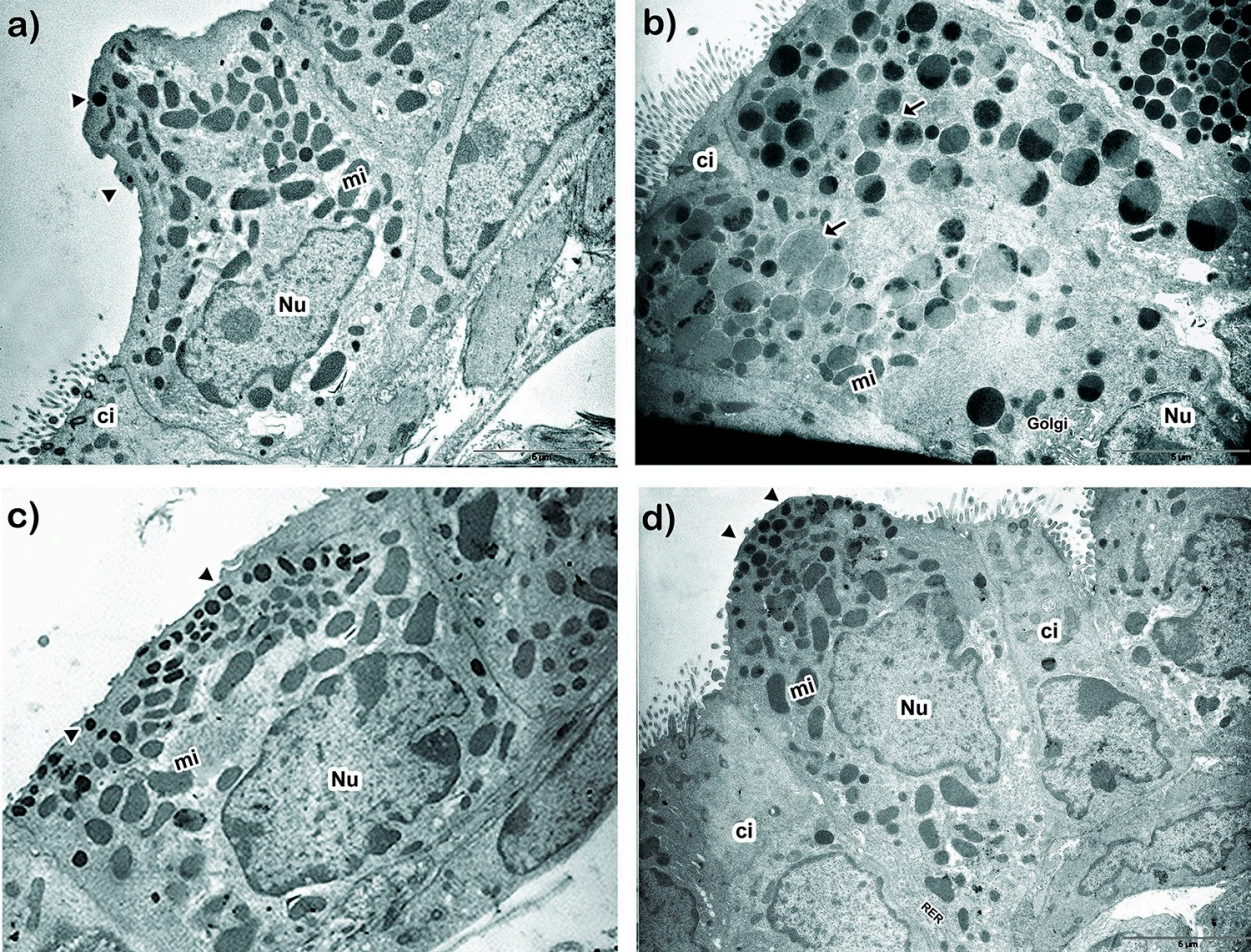
Club cell ultrastructural features. Representative electron micrograph images of CC morphology of PBSn (A), PBSn/OVA (B), LPSn (C) and LPSn/OVA (D) groups. Scale bar represents 5μm. Nu: nucleus, Mi: mitochondria, Ci: ciliated cells, Golgi: Golgi apparatus, RER: rough endoplasmic reticulum. Arrowheads: normal electron dense granules, arrows: electron lucid granules. All data are representative of at least 6 animals per group in 3 independent experiments.

In previous studies, we demonstrated that the ultrastructure of CC is a sensitive parameter of the airway allergic inflammation [54, 57]. For this reason, we studied the CC morphological profile in all groups by electron microscopy (Fig 5), which revealed the preservation of the typical cellular profile in PBSn mice, characterized by the presence of a dome-shape cupola and numerous polymorphic mitochondria in the cytoplasm, along with scarce spherical electron-dense secretory granules under the plasma membrane (Fig 5A). These parameters could also be seen in LPSn/OVA mice, differing only in an increased number of normal electron-dense granules along with a higher development of RER (Fig 5D). On the other hand, PBSn/OVA animals displayed a characteristic mucous cell metaplasia with a hypertrophied cytoplasm filled with numerous large electron-lucent secretory granules, slim mitochondria and abundant RER (Fig 5B). In control mice, which were only exposed to LPS, the CC also developed an increased number of electron-dense granules, as was shown in LPSn/OVA animals (Fig 5C). In this group, the evident reduction in their CC cupola was probably due to the repeated LPS intranasal instillation they received in neonatal life.

### Neonatal LPS stimulus promoted a long-lasting increase of innate mediators and Th2-immunomodulatory proteins in the bronchiolar epithelium

**Fig 6:**
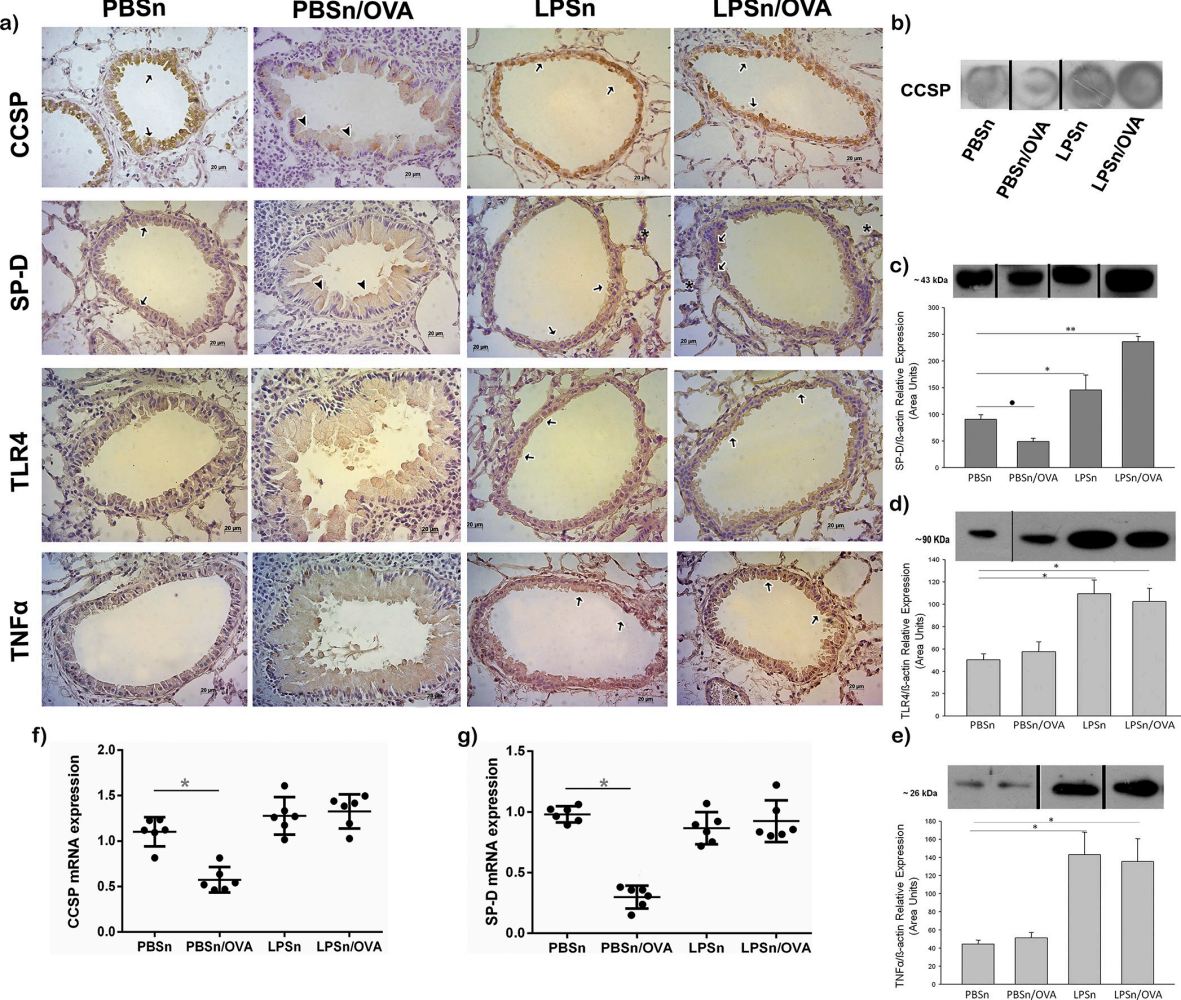
Club cell expression of host defense proteins and antimicrobial cytokines. (A) Immunostaining of CCSP, SP-D, TLR4 and TNFα performed on lung sections of all groups. Positive cells appear in brown against the blue counterstain of haematoxylin. Scale bars: 20μm. (B) Dot blot of CCSP expression in lung homogenates. Fragments of the same original blot were rearranged vertical black lines denote the spliced to remove irrelevant lanes. (C), (D) and (E) Western Blot of SP-D, TLR4 and TNFα lung content, respectively. Graph represents fold increase of the relative expression in lung homogenate by densitometric analysis. Fragments of the same original blot were rearranged; vertical black lines denote the spliced to remove irrelevant lanes. (F) and (G) CCSP and SP-D mRNA respectively expression by Real-Time PCR analysis. Graph represents fold increase expression in lung tissue homogenate. Data are represented as mean ±SEM, *p< 0.05 vs PBSn, **p<0.01 vs PBSn, ● p<0.05 vs PBSn. All data are representative of at least 6 animals per group in 3 independent experiments.

Next, we analyzed whether mucous metaplasia prevention by neonatal LPS treatment correlated with changes in the expression of the epithelial host defense mediators CCSP and SP-D as previously described (55). OVA-allergic inflammation induced a reduction in the immunoreactivity of CCSP and SP-D in CC of the PBSn/OVA group when compared to its control group (arrowhead in CCSP and SP-D panels in Fig 6A). In addition, for both LPSn and LPSn/OVA groups, a strong CCSP and SP-D immunolabeling was observed (arrows in CCSP and SP-D panels Fig 6A). These changes were also verified by immunoblotting (Fig 6B and 6C). However, neonatal LPS-instillation did not result in an increase in mRNA expression of CCSP or SP-D in LPSn or LPSn/OVA (Fig 6F and 6G, respectively). This may have been due to the stimulus for protein secretion provided by the allergen challenge in the LPSn/OVA group and/or to the contribution of SP-D of the Type II alveolar cells (asterisk in Fig 6A), which could explain the high SP-D content found by western blot analysis for these groups.

Regarding the microbial recognition receptor and cytokine response once activated, we found that, both TLR4 and TNFα increased their expression in both neonatal LPS-stimulated groups (Fig 6D and 6E and arrows in TLR4 and TNFα panels Fig 6E). Suggesting a specific response to the endotoxin in the bronchiolar epithelium that induced a persistent elevation of these defense molecules and seemed to be preserved in spite of allergen stimulus.

## Discussion

In the present work we demonstrate that neonatal LPS treatment triggers anti-allergic secretory products of the local airway epithelium that persist into adulthood. Among these products, CCSP and SP-D increased in the epithelial CC and lung tissue, together with the upregulation of TLR-4 and TNFα, which are related to innate host defenses. In correlation, CC skipped the mucous metaplasia pathway after an airborne allergen challenge, preserving their typical phenotype and reducing EGFR, mucins, and TSLP (a pro-Th2 cytokine) overexpression. Furthermore, under allergic stimulation, animals with neonatal LPS treatment exhibited normal breathing patterns and IL-4 and TSLP levels, but a lower eosinophilia as compared with control group. In contrast, in these mice, an increase in phagocytes and regulatory T cells (CD4+CD25+FOXP3+ and CD4+IL-10+), as well as in IL-12 and TNFα levels, was observed. These results reveal a possible novel preventative and therapeutic approach for asthma, focused on increasing airway resistance to environmental insults, rather than by suppressing the Th2 downstream inflammation once established. The finding of anti-allergic effects being associated with CCSP and SP-D is consistent with previous studies [19] [42, 43, 50–53]. Also, there is evidence that these proteins down-regulate type 2 differentiation of Th cells, inhibit allergen-activation of innate immune cells (eosinophils, basophils, and mast cells) and are reduced in both BAL and the serum of asthmatic individuals [49, 59, 60]. Previous reports from our laboratory revealed an interplay between the establishment of asthma and the reduction in CCSP and SP-D levels, which were later restored by Budesonide or Montelukast treatment in a mouse model [54].

Recently, we showed that the pre-treatment of adult mice with LPS before an allergic inflammation partially prevent CCSP and SP-D reduction in CC, with the increase of IL-4 levels, hindering airway hyperresponsiveness [57]. However, in the present work, we demonstrated that when endotoxin treatment is performed in neonatal life, it achieves more extensive asthma prevention in adulthood. This neonatal treatment not only avoided metaplastic changes in CC, but also preserved the mRNA levels of CCSP and SP-D. Moreover, the characteristic increase in IL-4 and respiratory distress after OVA challenge was limited. These results are similar to the blunting of a Th-2 allergic response and airway hyperresponsiveness reported by other authors using infant or pregnant mice and a microbial stimulus [13, 15, 17, 19].

In our study, a high dose of LPS was used, inducing a typical Th1 proinflammatory response, while suppressing the Th2-associated cytokine IL4 [17, 23]. As expected, an increase in TNFα and IL-12 was observed in both groups exposed to LPS. Nevertheless, a robust Th1 response was only seen in LPS-exposed animals as indicated by the high IFNγ levels, while in LPSn/OVA, the most important immunological change was the increased number of Tregs. In addition, whereas the LPSn/OVA group was the only one that attained a significant number of CD4+IL10+ cells, both groups (LPSn and LPSn/OVA) demonstrated a significantly higher ratio of IL10+ /CD4+ cells *ex vivo* compared to PBSn animals. Although the experimental design of our study cannot explain how a rise in IL-12 coexisted with a Treg response in the LPSn/OVA group, other authors have related the persistent increase in IL-12 cytokine with protection against a challenging infection [61, 62]. Investigations conducted by Gerhold et al. in 8-week-old Balb/c mice with systemic administration of an anti-IL-12 before LPS stimulus demonstrated that the reduction of an ulterior allergic inflammation occurs in an IL-12 dependent way [17]. Regarding the Treg response, Nguyen *et al*. previously described that TSLP directly impaired the function of pulmonary Treg cells obtained from allergic asthma patient [63], as indicated by a significant decrease in suppressive activity and IL-10 production, which was associated with the TSLP expression levels in BAL. Therefore, it is likely that the reduction in TSLP induced by LPS pretreatment in this study had the additional effect of restoring Treg cells.

In agreement with our results, experimental studies on neonatal Balb/c mice exposed to LPS with different protocols of sensitization and exposition to OVA demonstrated the occurrence of a response involving the expression of IL-10 and IFN-γ after re-exposition to allergen [15, 19]. Furthermore, Gerhold et al. demonstrated that LPS, in either prenatal or postnatal stimulation, induced a persistent elevation in soluble factors, such as CD14 and Lipopolysaccharide binding protein, and TLR4 mRNA expression in young mice [15]. More recently, the gene expression levels of innate and adaptive immunity essential markers in white blood cells in farmers' children were assessed in the multinational and prospective epidemiological study PARSIFAL [64]. This study compared farmers’ children to non-farmers’ children with respect to essential marker expression and the prevalence of asthma, with the authors showing an enhanced expression of innate immunity genes, such as IRAK-4 and RIPK1, as well as the regulatory molecules IL-10, TGF-beta, SOCS4, and IRAK-2 in farmers [64]. Although the correlation of Tregs and host defense molecules described was similar to our results, our findings pointed to evaluate the involvement of the epithelium.

As described above, several experimental and clinical studies have established a correlation between LPS pre-exposure and asthma phenotype abrogation, with our present study attempting to determine the changes occurring in this context in the pro-allergic cytokines secreted by the epithelium, such as TSLP. Our results demonstrated that neonatal exposition to LPS correlated with the abrogation of TSLP expression in the epithelium and BAL. Therefore, the diminution of TSLP could contribute to reduce the allergen-induced TLSP recruits dendritic cells that amplify the Th2 response and reduce Treg cell expansion [30]. In recent decades, several neonatal and pregnancy animal models have suggested that the transition from the quiescent Th2-polarized fetal immune phenotype towards the more active Th1-pattern of mature adaptive immunity is intrinsically slower in the atopic population, thereby increasing the risk of an allergen priming response against environmental antigens [21, 22] [65–70]. Hence, it would be important in future research to evaluate whether this early pro-inflammatory stimulus by LPS could cooperate with the progression of this transition.

LPS reduced the TSLP mRNA basal expression in the present study, which is consistent with its intrinsic capacity to counterbalance different pro- allergic actions, and blunted the subsequent overproduction of TSLP after OVA exposition. In a human bronchial epithelial cell line, Lin *et al* also demonstrated that LPS pre-treatment was able to reduce the induction of TSLP mRNA levels by means of a virus that causes neonatal respiratory disease [71]. Interestingly, in this study, the basal mRNA levels of the different signaling proteins involved in the TSLP overproduction were down-regulated only when a repeated LPS- preventive treatment was applied. In a process, these authors attributed this effect to the modulation in the expression of innate immunity signaling molecules of the airway epithelial cells that mitigate the allergic response.

The main contribution of the present study is to highlight the involvement of the bronchioalveolar epithelium in early microbial protection from an allergic disorder, a topic that still remains largely unaddressed. This was demonstrated by the stable changes observed in the expression of antiallergic and host defense factors by CC, as well as by the reduction to basal levels of potent epithelial-Th2 mediators, all of which were promoted by neonatal LPS-stimulation that further polarized the Treg response after an allergen exposition. Taken together, our results indicate that there are several anti-allergic dynamic mechanisms overlaid in the epithelium that may be favored in an adequate epidemiological environment.

## Supporting information

S1 File Original blot and gel images of the figure of the manuscript. The PDF file contains the uncropped as well as the minimally adjusted images supporting all blot and gel results.
(PDF)

S2 File Clinical score of respiratory distress after OVA-challenge: S2 Fig and S3 Table. S2 Fig: three independent physicians (including primary care physicians, a pneumologist and internists) performed the scoring using a double blind procedure. For the analysis of the clinical score data, we applied the Kruskal Wallis test. For all tests, a p<0.05 significance level was used. ***p<0.001 vs PBSn/OVA. S3 Table: Representative video recording of each group and score obtained.
(DOCX)

S3 File Representative video recordings of each group and scores obtained. Compressed Video Files. Representative video showing respiratory distress in the LPSn/OVA, PBSn, PBSn/OVA, LPSn groups.
(RAR)
