# Supplementary material for: Neonatal endotoxin stimulation is associated with a long-term bronchiolar epithelial expression of innate immune and anti-allergic markers that attenuates the allergic response
Source: PLoS One. 2020 May 7;15(5):e0226233. doi: 10.1371/journal.pone.0226233 (PMC7205282; doi:10.1371/journal.pone.0226233)

CCSP Dot Blot

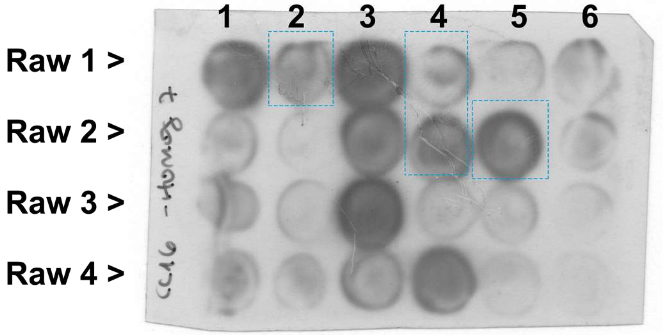

β-actin Dot Blot

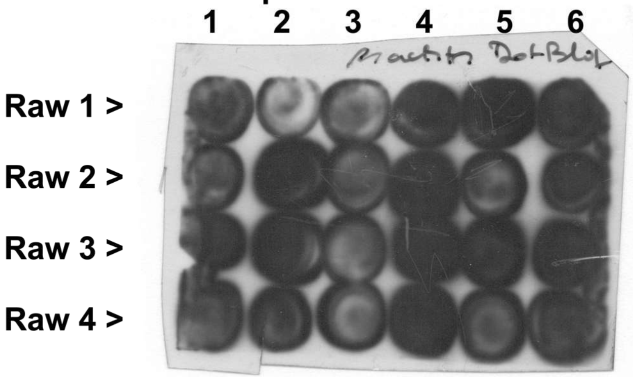

Raw 1
1.1 PBSn mice
1.2 PBSn mice
1.3 LPSn/OVA mice
1.4 PBSn/OVA mice
1.5 PBSn mice
1.6 PBSn mice
Raw 2
2.1 PBSn mice
2.2 PBSn/OVA mice
2.3 LPS n mice
2.4 LPS n mice
2.5 LPSn/OVA mice
2.6 PBSn mice
Raw 3
3.1 PBSn mice
3.2 PBSn/OVA mice
3.3 LPSn/OVA mice
3.4 PBSn/OVA mice
3.5 PBSn/OVA mice
3.6 PBSn/OVA mice
Raw 4
4.1 PBSn mice
4.2 PBSn mice
4.3 LPSn mice
4.4 LPSn/OVA mice
4.5 PBSn/OVA mice
4.6 PBSn/OVA mice

Image splitting

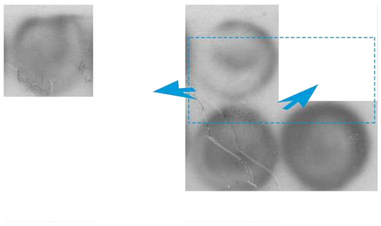

Rearranged figure

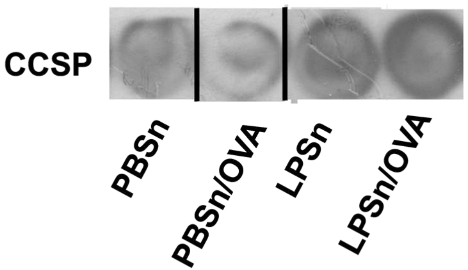

SP-D Immunoblot

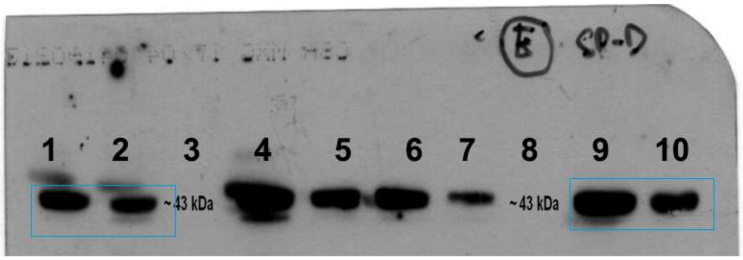

Lane
1 LPSn mice
2 PBSn/OVA mice
3 Molecular weight marker
4 LPSn/OVA mice X
5 PBSn/OVA mice X
6 LPSn mice X
7 PBSn/OVA mice X
8 Molecular weight marker X
9 LPSn/OVA mice
10 PBSn mice

β-actin Immunoblot

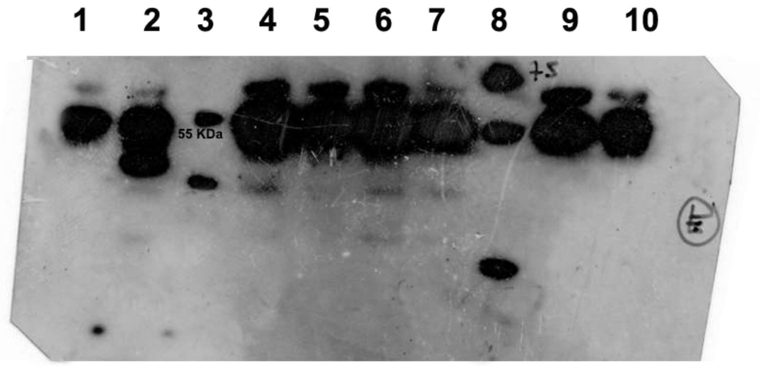

Image splitting

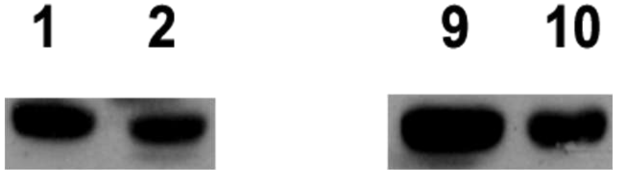

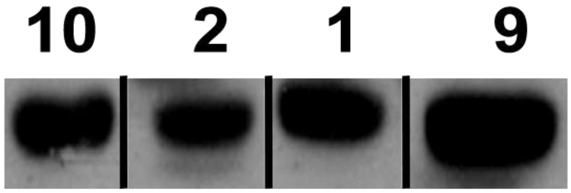

Rearranged figure

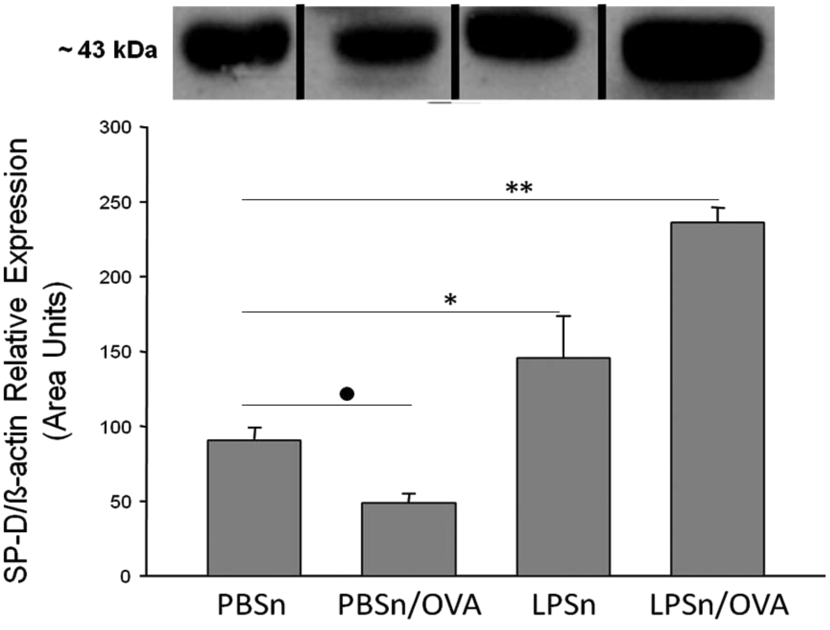

TLR4 Immunoblot

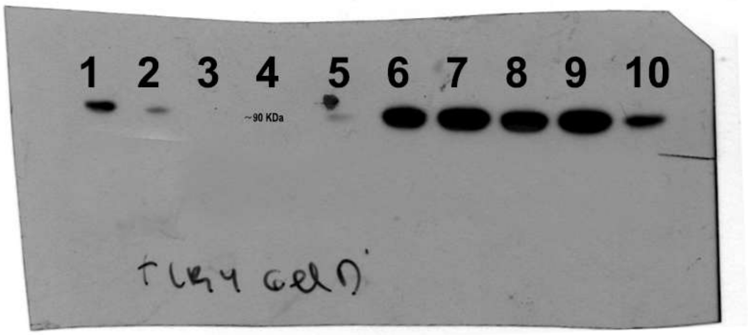

β-actin Immunoblot

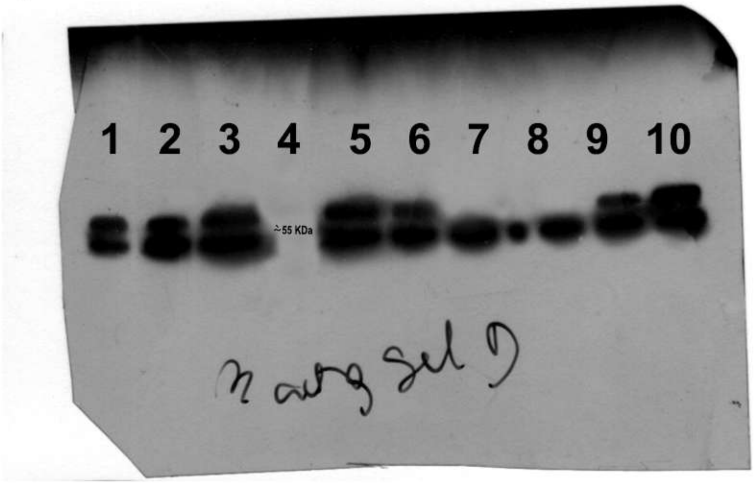

Lane
1 PBSn mice
2 PBSn/OVA mice X
3 PBSn mice X
4 Molecular weight marker
5 PBSn/OVA mice X
6 LPSn/OVA mice X
7 LPSn mice X
8 LPSn/OVA mice
9 LPSn mice
10 PBSn/OVA mice

Image splitting

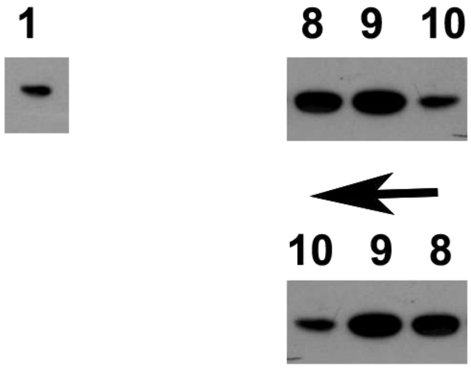

Rearranged figure

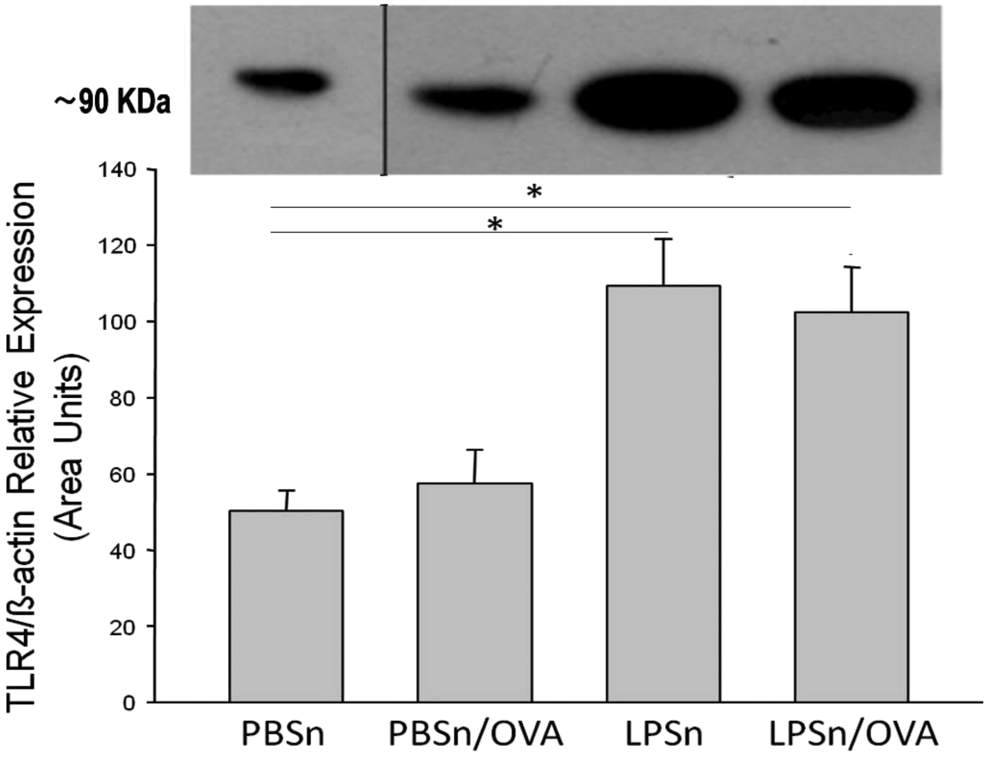

TNFα Immunoblot

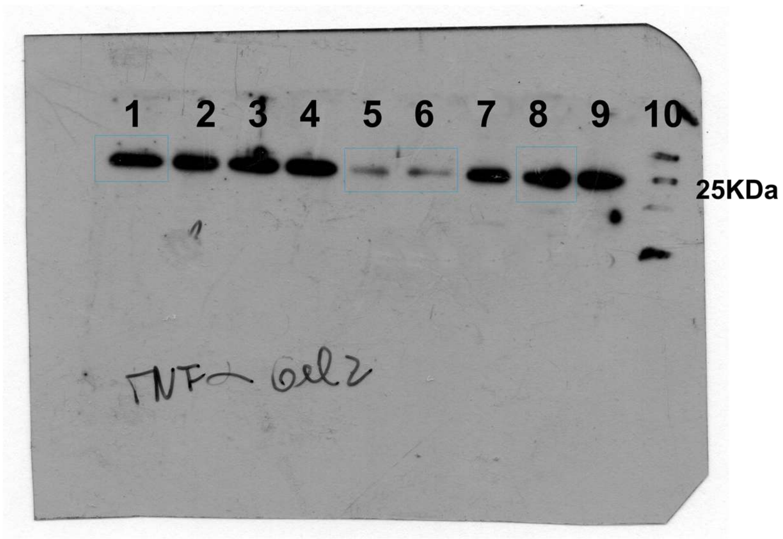

Lane

1 LPSn mice
2 LPSn mice X
3 LPSn mice X
4 LPSn mice X
5 PBSn mice
6 PBSn/OVA mice
7 LPSn/OVAmice X
8 LPSn/OVA mice
9 LPSn/OVA mice X
10 Molecular weight marker

β-actin Immunoblot

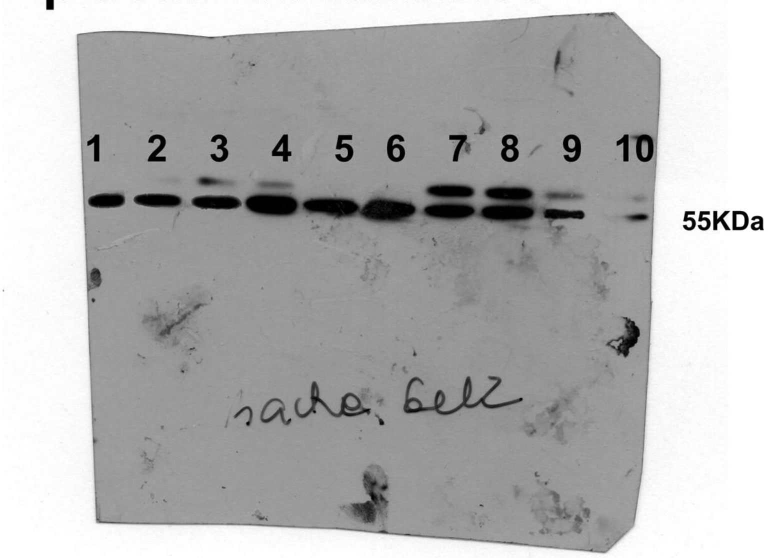

Image splitting

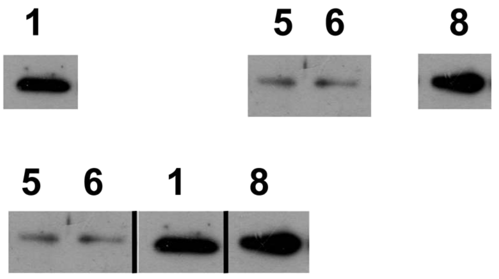

Rearranged figure

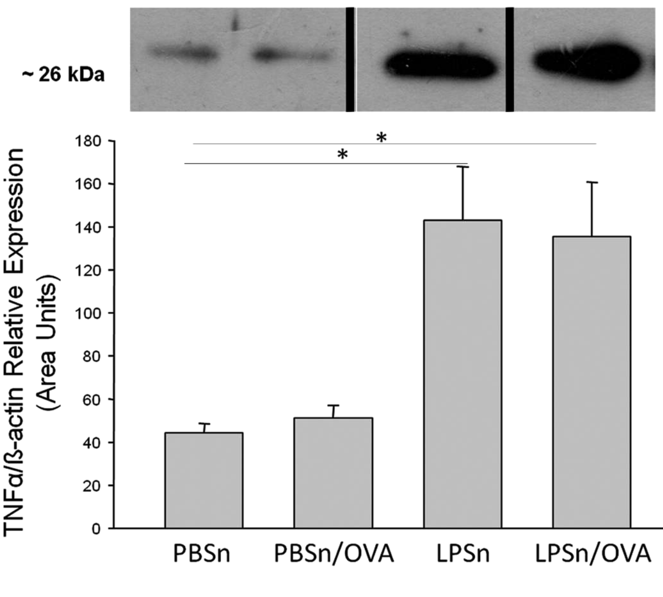

Supplement: S1 File — The PDF file contains the uncropped as well as the minimally adjusted images supporting all blot and gel results. (PDF) [file pone.0226233.s001.pdf]
