# Supplementary material for: Neonatal endotoxin stimulation is associated with a long-term bronchiolar epithelial expression of innate immune and anti-allergic markers that attenuates the allergic response
Source: PLoS One. 2020 May 7;15(5):e0226233. doi: 10.1371/journal.pone.0226233 (PMC7205282; doi:10.1371/journal.pone.0226233)
Supplement: S2 File — S2 Fig: three independent physicians (including primary care physicians, a pneumologist and internists) performed the scoring using a double blind procedure. For the analysis of the clinical score data, we applied the Kruskal Wallis test. For all tests, a p<0.05 significance level was used. ***p<0.001 vs PBSn/OVA. S3 Table: Representative video recording of each group and score obtained. (DOCX) [file pone.0226233.s002.docx]

**Clinical score of respiratory distress after OVA-challenge**

A clinical score system for assessing the degree of respiratory distress was applied to mice (n=5) in all groups, using a video recording of the breathing patterns exhibited during the first minute after OVA/saline instillation at days 7-10 of the challenge for the three different protocols. Values were assigned by the adaption of Wood's asthma score (S2 Fig) for increasing signs of respiratory distress (S3 Table). This data for the groups of mice is shown in the S4 -8 Videos


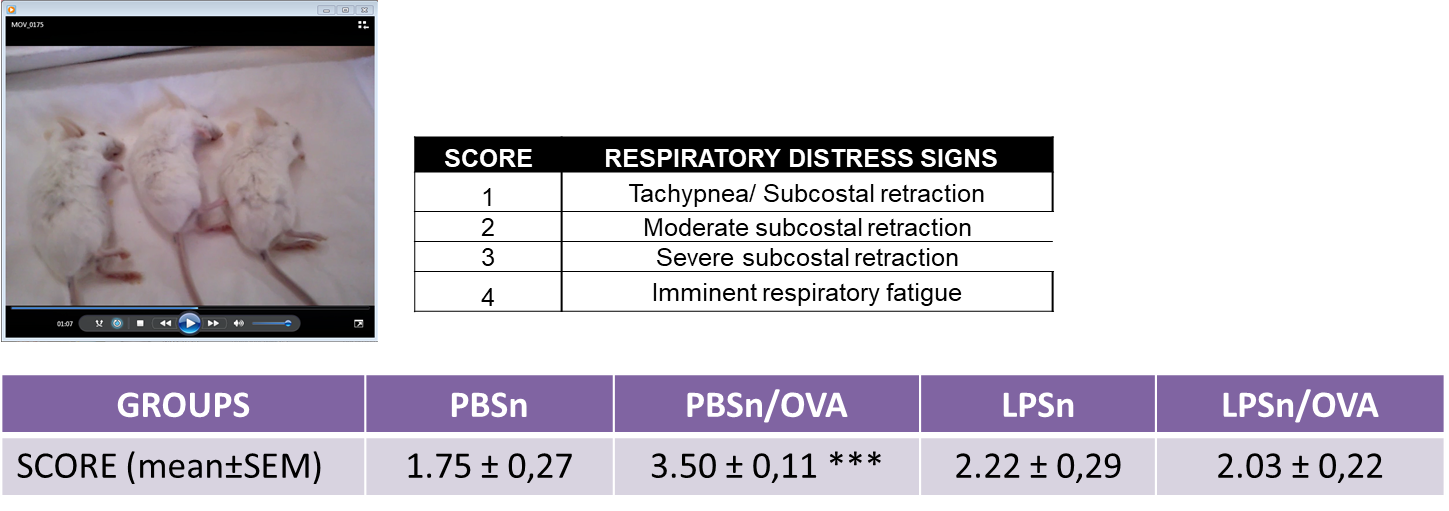


S2 Fig. The scoring was performed using a double blind procedure by three independent physicians (including primary care physicians, a pneumologist and internists) Supplementary table 1. For the analysis of the clinical score data, we applied the Kruskal Wallis test. For all tests, a p<0.05 significance level was used. ***p<0.001 vs PBSn/OVA

S3 table Representative video recording of each group and score obtained

|  |  | **Score obtained** | | |
| --- | --- | --- | --- | --- |
| **Group** | **Record label** | **Operator 1** | **Operator 2** | **Operator 3** |
| LPSn/OVA | MOV-104404 | 1 | 2 | 1 |
| PBSn | MOV-0172 | 1 | 1 | 1 |
| PBSn/OVA | MOV_0175 | 4 | 3 | 3 |
| LPSn | MOV_1504 | 1 | 2 | 1 |
