# Supplementary material for: Neonatal endotoxin stimulation is associated with a long-term bronchiolar epithelial expression of innate immune and anti-allergic markers that attenuates the allergic response
Source: PLoS One. 2020 May 7;15(5):e0226233. doi: 10.1371/journal.pone.0226233 (PMC7205282; doi:10.1371/journal.pone.0226233)
Supplement: Attachment — Submitted filename: Response to reviewers.docx [file pone.0226233.s004.docx]

Additional Editor Comments

**1. The manuscript writing was improved as observed in Editor comments**

**2. Abstract was modified in order to better focus on manuscript’s findings.**

**3. Full definitions for all abbreviations should be provided upon their first mentioning in the manuscript’s text (f.e. CCSP, SPD, etc.).**

- Manuscript text was changed attending these three editor comments

4. **Clarify what Helsinki convention has to do with animal housing and caring and what local country and institutional regulations were followed during the experiments involved animals.**

- We realized that there was a mistake because Helsinki convention is not related to animal caring. We replaced this affirmation by the correct one: “Animal care and experiments were conducted following the recommendations of the International Guiding Principles for Biomedical Research Involving Animals and approved by the local Ethical Committee (CICUAL Commission for Care and Use of Laboratory Animals) of the School of Medicine, National University of Córdoba)”.

**5. Low dose and high dose of LPS have different effects on asthma outcome. Please discuss relevant published literature and explain why such dose of LPS was selected.**

- In Introduction of the revised version we now discuss the differential effect of low vs high dose of LPS, and introduced the following literature:

Eisenbarth S. C., Piggott D. A., Huleatt J. W., Visintin I., Herrick C. A., Bottomly K. J. *Lipopolysaccharide-enhanced, toll-like receptor 4-dependent T helper cell type 2 responses to inhaled antigen*; Exp. Med. 2002;196:1645–1651.

Simpson A, Martinez FD; The role of lipopolysaccharide in the development of atopy in humans; Clin Exp Allergy; 40: 209-23;2010

Nigo YI, Yamashita M, Hirahara K, Shinnakasu R, Inami M, et al.; Regulation of allergic airway inflammation through Toll-like receptor 4-mediated modification of mast cell function; Proc Natl Acad Sci U S A; 103: 2286-91;2006

**6. 0.1 ml of OVA grade VI does not give us exact allergen concentration. Clarify.**

- The text and figure 1 were modified as following

*Allergen sensitization in weaned animals:* At the age of 4 weeks, all female animals were selected and sensitized by subsequent i.p injections of 0.1 ml	of Ovalbumin (OVA) grade VI (100 µg/100 μl, Sigma-Aldrich) absorbed to 1 mg of inject Alum (Pierce Rockford, USA) on the 4º and 6º weeks of life.

**7. Reference previously described lung morphology analysis accordingly. Similar for AB-PAS stain.**

We introduced the following literature to reference lung morphology and AB-PAS stain:

Roth FD, Quintar AA, Uribe Echevarria EM, Torres AI, Aoki A, Maldonado CA. Budesonide effects on Clara cell under normal and allergic inflammatory condition. Histochem Cell Biol. 2007;127(1):55-68

**8. Were samples equalized for a protein level before Western blot?**

- Yes, it does (lines 219-222 in Materials and methods). We also specify that the expression of mouse anti-βactin (ACTB) was used as an internal control to confirm equivalent total protein loading (lines 232-233).

**9. Specify software used for statistical analysis.**

- Statistical tests were performed using the InfoStat (Faculty of Agricultural Sciences, National University of Córdoba, Córdoba, Argentina) statistical program (text introduced in revised version)

**10. Please explain why LPS treatment increases IFNgamma but does not affect IL-4 levels in BAL.**

- As we now clarified in Introduction, it has been described that the exposure to high-level LPS with antigens resulted in increased antigen-specific T herlper (Th) 1 responses, whereas a low dose of LPS resulted in Th2 sensitization with LPS acting as a Th2 adjuvant (Eisenbarth S. C., Piggott D. A., Huleatt J. W., Visintin I., Herrick C. A., Bottomly K. J. Exp. Med. 2002;196:1645–1651). Therefore it is expected that changes induced by neonatal-LPS increases IFNgamma but does not affect IL-4 levels in BAL after asthma experimental protocol.

**11**. **Figure 2. Specify where cytokine levels were measured (BAL, serum, cell culture supernatants, etc.). Although it is stated in a title, first sentence in figure legend is missing this point.**

- This modification was done in the revised version

**11. Panel d. It looks like OVA treatment induces Treg cells in BAL. Strongly. Now over 24% of CD4+ T cells co-express CD25 and Foxp3. Show a panel with absolute numbers of Treg cells for all experimental conditions. Should you focus on Foxp3+IL-10+ cells in this figure?**

- The panel with absolute Treg number was introduced

**12. Figure 5, panel a. Incorporate arrows to point on where to look for described results.**

- This modification was introduced in the revised version

| Comments Reviewer 1:  1. **Lines 114-115: Please rephrase.**  This was corrected as suggested by the reviewer.  2. **Lines 118-120: Please rephrase**  This was corrected as suggested by the reviewer.  3. **Lines 305-306: 4-6 weeks old mice are not really adult mice. Please adapt that in the whole text.**  As stated in the experimental design and in the timeline diagram in Fig 1, mice reached 8-9 weeks old. According to bibliography, 8-12 weeks old in mice physiologically resembles human adulthood (http://dx.doi.org/10.1016/j.lfs.2015.10.025). However, to be more accurate, we introduced the relative ages of mice in the different steps in “experimental design” section, indicating in allergen challenge the starting and ending age of Balb/c mice. To meet the journal recommendations, this protocols has been uploaded to protocols.io (dx.doi.org/10.17504/protocols.io.bbx2ipqe)  **4. Line 307: there is a ‘t’ missing in the word ‘first’**.  This was corrected as suggested by the reviewer.  **5. Figure legends: Please indicate the number of experiments and the number of animals used in each experiment**.  This was corrected following the reviewer's concern.  **6. Figures: Please don’t show bar graphs. Dot blots would be more suitable. And please indicate the significance between specific groups in the graph not only in the legend.**  Following this comment, Fig 5 (Fig 6 in the revised manuscript) we changed bar graph for Dot blot spots. Moreover, all graphs of the manuscript were modified and the significance among groups were indicated.  7. **Figure 1b: It would increase the readability of graph if the cell types of the different groups would be next to each other. Like this, the difference in eosinophils between PBS/OVA and LPS/OVA is difficult to observe. And please don’t show discontinuous axes.**  This was corrected as suggested by the reviewer. The figure is now easier to interpret.  **8. Lines 316: It should be Fig 1b.**  In the present version of the manuscript, it is Figure 2B  **9. Figure 1: It would be interesting to see if there is a difference in cytokines like IL-5, IL-13, IFN-gamma or IL-10.**  Regarding this concern, we have already evaluated the levels of IFN-gamma and, they are shown Fig 2A. We have also measured IL-10 in BAL using a commercially available sandwich ELISA kit (#14-7101-67, eBioscience, San Diego, USA) but this cytokine could not be detected. This was probably due to the kit sensitivity (30 pg/ml) or to low concentrations of the cytokine in the BAL. According to the bibliography (doi: 10.1007/978-1-4939-1161-5_5), a more efficient system for cytokine detection is by using a protein transport inhibitor in combination with intracellular staining, with the revealing being performed by immunofluorescence or flow cytometry. In fact, for intracellular IL10 detection, this technique achieved good results as it is shown in the present version of the manuscript (Fig 3F) (dx.doi.org/10.17504/protocols.io.bdxzi7p6).  The Th2-related cytokines IL-4 and TSLP have also been determined based on the primary role of IL-4 as well as the expression of IL4R in club cells (92-93 line introduction) and on the epithelial involvement of TSLP (78-81 line introduction).  **10. Line 332: the term ‘airway’s milieu’ should be specified clearer.**  “airway´s milieu” was replaced by “milieu within the airways” and a introduction sentence expresses the reason for the analysis was added – Lines 324-328.  **11. Lines 337-341: This part should be explained a bit deeper. Where the cells only stained for IL-10 or IL-10 plus Foxp-3/CD25? Why was the ratio between IL-10 and CD4 cells was calculated?**  Referring this comment, cells were stained for Foxp-3/CD25 and IL-10, but following different techniques, flow cytometry and immunofluorescence respectively. By immunofluorescence, the ratio CD4/IL-10was calculated in order to evaluate only IL-10-producing lymphocytes, excluding macrophages and neutrophils. In the revised version, we have introduced a second table with the % of CD25+ cells expressing Foxp-3 by flow cytometry.  **12. Line 357-358: The mentioned ‘specific pro-inflammatory’ should be specified more clearly. At the end of the sentence, there is a comma instead of a point.**  In response to this, we suppressed the term and added two new sentences with concepts and clarifications related to the nature of the molecules we are analyzing. Lines 291-293 and 354-356 of Results  **13. Figure 3. It would be nice to see the quantification not only for the AB-PAS staining but also for TSLP and pEGFR.**    Following this suggestion, we performed a densitometric analysis to quantify TSLP expression by using ImageJ on immunostained sections. A panel graph was also added in Fig 3 (Now Figure 4C) as well as a paragraph in Material and Methods section explaining it. Regarding pEGFR, as its immunolabeling is restricted to the apical cytoplasm of CC after OVA challenge, densitometry would not reflect the actual expression or the changes in pEGFR among the groups. |  |  |
| --- | --- | --- |
