# Supplementary material for: Neonatal endotoxin stimulation is associated with a long-term bronchiolar epithelial expression of innate immune and anti-allergic markers that attenuates the allergic response
Source: PLoS One. 2020 May 7;15(5):e0226233. doi: 10.1371/journal.pone.0226233 (PMC7205282; doi:10.1371/journal.pone.0226233)
Supplement: Attachment — Submitted filename: Response to editor.docx [file pone.0226233.s005.docx]

Responses to Academic Editor:

1. **Airway challenge in puberty and adulthood? Is this an appropriate statement for mice?**

Following bibliography and usual terminology, both terms are appropriate for age determination of laboratory mice (Revise in <http://dx.doi.org/10.1016/j.lfs.2015.10.025>)

2. **Line 232. Mouse anti-b-actin expression is actually a mouse b-actin detection with anti-b-actin Ab**

This was corrected as suggested.

3. **Are website links to the protocol used acceptable for the journal? Like shown in lines 143, 177, 202, 215, 243, 271, and 276?**

During the previous revision process we have already uploaded our laboratory protocols to [protocols.io](http://protocols.io/). The website links cited in the Methods section were, actually, the assigned identifier (DOI) provided by protocols.io as submission-guidelines requirements.

| **Laboratory protocol** | **DOI** | **Line** |
| --- | --- | --- |
| Experimental design | http://dx.doi.org/10.17504/protocols.io.bdwwi7fe | 141 |
| Immunohistochemical analysis of lung tissue | http://dx.doi.org/10.17504/protocols.io.bdw9i7h6 | 175 |
| Immunofluorescence | http://dx.doi.org/10.17504/protocols.io.bdxzi7p6 | 199 |
| Flow cytometry | http://dx.doi.org/10.17504/protocols.io.bdx3i7qn | 212 |
| Dot Blot Analysis | http://dx.doi.org/10.17504/protocols.io.bbyhipt6 | 241 |
| RNA isolation and gene expression analysis | http://dx.doi.org/10.17504/protocols.io.bdx5i7q6 | 268 |
| Clinical score assessment of the degree of respiratory distress | http://dx.doi.org/10.17504/protocols.io.bbymipu6 | 273 |

4. **IFNg, PCR, and many others are standard abbreviations for the journal. No full definition is needed. TSLP ROS, PE, and PerCP probably too. Please correct. Note, that upon their first mentioning in the manuscript text, which is in the abstract, no full definition is provided.**

The abbreviations were added to the abstract and we revised the manuscript to keep them a minimum.
