# Supplementary material for: Neonatal endotoxin stimulation is associated with a long-term bronchiolar epithelial expression of innate immune and anti-allergic markers that attenuates the allergic response
Source: PLoS One. 2020 May 7;15(5):e0226233. doi: 10.1371/journal.pone.0226233 (PMC7205282; doi:10.1371/journal.pone.0226233)
Supplement: Peer review history

# Peer review: Decision Letter 0

**DOI:** 10.1371/journal.pone.0226233.r001

**Authors:** Svetlana P. Chapoval

13 Jan 2020

PONE-D-19-32052

Neonatal endotoxin stimulation was associated with long-term innate immune markers and an anti-allergic response in bronchiolar epithelium even after allergen challenge

PLOS ONE

Dear Dr. Luciana Noemi Garcia,

Thank you for submitting your manuscript to PLOS ONE. After careful consideration, we feel that it has merit but does not fully meet PLOS ONE’s publication criteria as it currently stands. Therefore, we invite you to submit a revised version of the manuscript that addresses the points raised during the review process.

We would appreciate receiving your revised manuscript by September 13, 2020. When you are ready to submit your revision, log on to https://www.editorialmanager.com/pone/ and select the 'Submissions Needing Revision' folder to locate your manuscript file.

If you would like to make changes to your financial disclosure, please include your updated statement in your cover letter.

To enhance the reproducibility of your results, we recommend that if applicable you deposit your laboratory protocols in protocols.io, where a protocol can be assigned its own identifier (DOI) such that it can be cited independently in the future. For instructions see: http://journals.plos.org/plosone/s/submission-guidelines#loc-laboratory-protocols

Please include the following items when submitting your revised manuscript:

- A rebuttal letter that responds to each point raised by the academic editor and reviewer(s). This letter should be uploaded as separate file and labeled 'Response to Reviewers'.
- A marked-up copy of your manuscript that highlights changes made to the original version. This file should be uploaded as separate file and labeled 'Revised Manuscript with Track Changes'.
- An unmarked version of your revised paper without tracked changes. This file should be uploaded as separate file and labeled 'Manuscript'.

Please note while forming your response, if your article is accepted, you may have the opportunity to make the peer review history publicly available. The record will include editor decision letters (with reviews) and your responses to reviewer comments. If eligible, we will contact you to opt in or out.

We look forward to receiving your revised manuscript.

Kind regards,

Svetlana P. Chapoval

Academic Editor

PLOS ONE

Additional Editor Comments (must be addressed in revised manuscript):

1. Manuscript requires a major editorial work.

2. Abstract should focus on manuscript’s findings.

3. Full definitions for all abbreviations should be provided upon their first mentioning in the manuscript’s text (f.e. CCSP, SPD, etc.).

4. Clarify what Helsinki convention has to do with animal housing and caring and what local country and institutional regulations were followed during the experiments involved animals.

5. Low dose and high dose of LPS have different effects on asthma outcome. Please discuss relevant published literature and explain why such dose of LPS was selected.

6. 0.1 ml of OVA grade VI does not give us exact allergen concentration. Clarify.

7. Reference previously described lung morphology analysis accordingly. Similar for AB-PAS stain.

8. Were samples equalized for a protein level before Western blot?

9. Specify a software used for statistical analysis.

10. Please explain why LPS treatment increases IFNgamma but does not affect IL-4 levels in BAL.

11. Figure 2. Specify where cytokine levels were measured (BAL, serum, cell culture supernatants, etc.). Although it is stated in a title, first sentence in figure legend is missing this point. Panel d. It looks like OVA treatment induces Treg cells in BAL. Strongly. Now over 24% of CD4+ T cells co-express CD25 and Foxp3. Show a panel with absolute numbers of Treg cells for all experimental conditions. Should you focus on Foxp3+IL-10+ cells in this figure?

12. Figure 5, panel a. Incorporate arrows to point on where to look for described results.

Journal requirements:

When submitting your revision, we need you to address these additional requirements.

1. Please ensure that your manuscript meets PLOS ONE's style requirements, including those for file naming. The PLOS ONE style templates can be found at

http://www.journals.plos.org/plosone/s/file?id=wjVg/PLOSOne_formatting_sample_main_body.pdf and http://www.journals.plos.org/plosone/s/file?id=ba62/PLOSOne_formatting_sample_title_authors_affiliations.pdf

2. Thank you for including your ethics statement:

"Animal care and experiments were conducted following the recommendations of the International Guiding Principles for Biomedical Research Involving Animals and by the local CICUAL Ethical Committee. The experiments were conducted following the recommendations of Helsinki convention, and anaesthetic agents were provided during the different procedures. During airway challenge, animals were lightly anesthetized by inhaled isoflurane before the delivery dropwise to the nares of the volume using a pipetman; wich allows a quicker recovery time. Meanwhile for Bronchoalveolar lavage and lung collection mice were anesthetized (i.p.) with a mixture of ketamine (60 mg/kg) and xylazine (15 mg/kg) and immediately sacrificed by exsanguination."

a. Please amend your current ethics statement to confirm that your named ethics committee specifically approved this study.

b. Once you have amended this/these statement(s) in the Methods section of the manuscript, please add the same text to the “Ethics Statement” field of the submission form (via “Edit Submission”).

For additional information about PLOS ONE submissions requirements for ethics oversight of animal work, please refer to http://journals.plos.org/plosone/s/submission-guidelines#loc-animal-research

3. PLOS ONE now requires that authors provide the original uncropped and unadjusted images underlying all blot or gel results reported in a submission’s figures or Supporting Information files. This policy and the journal’s other requirements for blot/gel reporting and figure preparation are described in detail at https://journals.plos.org/plosone/s/figures#loc-blot-and-gel-reporting-requirements and https://journals.plos.org/plosone/s/figures#loc-preparing-figures-from-image-files. When you submit your revised manuscript, please ensure that your figures adhere fully to these guidelines and provide the original underlying images for all blot or gel data reported in your submission. See the following link for instructions on providing the original image data: https://journals.plos.org/plosone/s/figures#loc-original-images-for-blots-and-gels.

In your cover letter, please note whether your blot/gel image data are in Supporting Information or posted at a public data repository, provide the repository URL if relevant, and provide specific details as to which raw blot/gel images, if any, are not available. Email us at plosone@plos.org if you have any questions.

4. Please amend either the title on the online submission form (via Edit Submission) or the title in the manuscript so that they are identical.

5. Please amend either the abstract on the online submission form (via Edit Submission) or the abstract in the manuscript so that they are identical.

6. Please include captions for your Supporting Information files at the end of your manuscript, and update any in-text citations to match accordingly. Please see our Supporting Information guidelines for more information: http://journals.plos.org/plosone/s/supporting-information

Reviewers' comments:

Reviewer's Responses to Questions

**Comments to the Author**

1. Is the manuscript technically sound, and do the data support the conclusions?

The manuscript must describe a technically sound piece of scientific research with data that supports the conclusions. Experiments must have been conducted rigorously, with appropriate controls, replication, and sample sizes. The conclusions must be drawn appropriately based on the data presented. 

Reviewer #1: Yes

**********

2. Has the statistical analysis been performed appropriately and rigorously? 

Reviewer #1: Yes

**********

3. Have the authors made all data underlying the findings in their manuscript fully available?

The PLOS Data policy requires authors to make all data underlying the findings described in their manuscript fully available without restriction, with rare exception (please refer to the Data Availability Statement in the manuscript PDF file). The data should be provided as part of the manuscript or its supporting information, or deposited to a public repository. For example, in addition to summary statistics, the data points behind means, medians and variance measures should be available. If there are restrictions on publicly sharing data—e.g. participant privacy or use of data from a third party—those must be specified.

Reviewer #1: Yes

**********

4. Is the manuscript presented in an intelligible fashion and written in standard English?

PLOS ONE does not copyedit accepted manuscripts, so the language in submitted articles must be clear, correct, and unambiguous. Any typographical or grammatical errors should be corrected at revision, so please note any specific errors here.

Reviewer #1: Yes

**********

5. Review Comments to the Author

Please use the space provided to explain your answers to the questions above. You may also include additional comments for the author, including concerns about dual publication, research ethics, or publication ethics. (Please upload your review as an attachment if it exceeds 20,000 characters)

Reviewer #1: The study presents an asthma preventive effect later in life of neonatal application of LPS in mice. The study shows that neonatal LPS exposure reduced the severity of the disease by a reduction of inflammatory cells in BALs, a breathing score, cytokines, increased regulatory T cells and by several effects on epithelial cells.

The study is accurately made, well analyzed and certainly of interest because it adds another piece to the understanding of how LPS might protect from the development of allergic diseases. A limitation is the missing of data about airway hyperresponsiveness to methacholine.

Comments:

1. Lines 114-115: Please rephrase

2. Lines 118-120: Please rephrase

3. Lines 305-306: 4-6 weeks old mice are not really adult mice. Please adapt that in the whole text.

4. Line 307: there is a ‘t’ missing in the word ‘first’.

5. Figure legends: Please indicate the number of experiments and the number of animals used in each experiment.

6. Figures: Please don’t show bar graphs. Dot blots would be more suitable. And please indicate the significance between specific groups in the graph not only in the legend.

7. Figure 1b: It would increase the readability of graph if the cell types of the different groups would be next to each other. Like this, the difference in eosinophils between PBS/OVA and LPS/OVA is difficult to observe. And please don’t show discontinuous axes.

8. Lines 316: It should be Fig 1b.

9. Figure 1: It would be interesting to see if there is a difference in cytokines like IL-5, IL-13, IFN-gamma or IL-10.

10. Line 332: the term ‘airway’s milieu’ should be specified clearer.

11. Lines 337-341: This part should be explained a bit deeper. Where the cells only stained for IL-10 or IL-10 plus Foxp-3/CD25? Why was the ratio between IL-10 and CD4 cells was calculated?

12. Line 357-358: The mentioned ‘specific pro-inflammatory mediators’ should be specified more clearly. At the end of the sentence, there is a comma instead of a point.

13. Figure 3. It would be nice to see the quantification not only for the AB-PAS staining but also for TSLP and pEGFR.

**********

6. PLOS authors have the option to publish the peer review history of their article (what does this mean?). If published, this will include your full peer review and any attached files.

If you choose “no”, your identity will remain anonymous but your review may still be made public.

**Do you want your identity to be public for this peer review?** For information about this choice, including consent withdrawal, please see our Privacy Policy.

Reviewer #1: Yes: Remo Frei, Department for BioMedical Research, University of Bern, Switzerland

[NOTE: If reviewer comments were submitted as an attachment file, they will be attached to this email and accessible via the submission site. Please log into your account, locate the manuscript record, and check for the action link "View Attachments". If this link does not appear, there are no attachment files to be viewed.]

While revising your submission, please upload your figure files to the Preflight Analysis and Conversion Engine (PACE) digital diagnostic tool, https://pacev2.apexcovantage.com/. PACE helps ensure that figures meet PLOS requirements. To use PACE, you must first register as a user. Registration is free. Then, login and navigate to the UPLOAD tab, where you will find detailed instructions on how to use the tool. If you encounter any issues or have any questions when using PACE, please email us at figures@plos.org. Please note that Supporting Information files do not need this step.

# Author comment: Author response to Decision Letter 0

**DOI:** 10.1371/journal.pone.0226233.r002

31 Mar 2020

Additional Editor Comments

1. The manuscript writing was improved as observed in Editor comments

2. Abstract was modified in order to better focus on manuscript’s findings.

3. Full definitions for all abbreviations should be provided upon their first mentioning in the manuscript’s text (f.e. CCSP, SPD, etc.). 

✔ Manuscript text was changed attending these three editor comments

4. Clarify what Helsinki convention has to do with animal housing and caring and what local country and institutional regulations were followed during the experiments involved animals.

✔ We realized that there was a mistake because Helsinki convention is not related to animal caring. We replaced this affirmation by the correct one: “Animal care and experiments were conducted following the recommendations of the International Guiding Principles for Biomedical Research Involving Animals and approved by the local Ethical Committee (CICUAL Commission for Care and Use of Laboratory Animals) of the School of Medicine, National University of Córdoba)”. 

5. Low dose and high dose of LPS have different effects on asthma outcome. Please discuss relevant published literature and explain why such dose of LPS was selected.

✔ In Introduction of the revised version we now discuss the differential effect of low vs high dose of LPS, and introduced the following literature: 

Eisenbarth S. C., Piggott D. A., Huleatt J. W., Visintin I., Herrick C. A., Bottomly K. J. Lipopolysaccharide-enhanced, toll-like receptor 4-dependent T helper cell type 2 responses to inhaled antigen; Exp. Med. 2002;196:1645–1651.

 Simpson A, Martinez FD; The role of lipopolysaccharide in the development of atopy in humans; Clin Exp Allergy; 40: 209-23;2010

 Nigo YI, Yamashita M, Hirahara K, Shinnakasu R, Inami M, et al.; Regulation of allergic airway inflammation through Toll-like receptor 4-mediated modification of mast cell function; Proc Natl Acad Sci U S A; 103: 2286-91;2006

6. 0.1 ml of OVA grade VI does not give us exact allergen concentration. Clarify.

✔ The text and figure 1 were modified as following

 Allergen sensitization in weaned animals: At the age of 4 weeks, all female animals were selected and sensitized by subsequent i.p injections of 0.1 ml of Ovalbumin (OVA) grade VI (100 µg/100 μl, Sigma-Aldrich) absorbed to 1 mg of inject Alum (Pierce Rockford, USA) on the 4º and 6º weeks of life.

7. Reference previously described lung morphology analysis accordingly. Similar for AB-PAS stain. 

 We introduced the following literature to reference lung morphology and AB-PAS stain:

Roth FD, Quintar AA, Uribe Echevarria EM, Torres AI, Aoki A, Maldonado CA. Budesonide effects on Clara cell under normal and allergic inflammatory condition. Histochem Cell Biol. 2007;127(1):55-68

8. Were samples equalized for a protein level before Western blot? 

✔ Yes, it does (lines 219-222 in Materials and methods). We also specify that the expression of mouse anti-βactin (ACTB) was used as an internal control to confirm equivalent total protein loading (lines 232-233).

9. Specify software used for statistical analysis. 

✔ Statistical tests were performed using the InfoStat (Faculty of Agricultural Sciences, National University of Córdoba, Córdoba, Argentina) statistical program (text introduced in revised version) 

10. Please explain why LPS treatment increases IFNgamma but does not affect IL-4 levels in BAL.

✔ As we now clarified in Introduction, it has been described that the exposure to high-level LPS with antigens resulted in increased antigen-specific T herlper (Th) 1 responses, whereas a low dose of LPS resulted in Th2 sensitization with LPS acting as a Th2 adjuvant (Eisenbarth S. C., Piggott D. A., Huleatt J. W., Visintin I., Herrick C. A., Bottomly K. J. Exp. Med. 2002;196:1645–1651). Therefore it is expected that changes induced by neonatal-LPS increases IFNgamma but does not affect IL-4 levels in BAL after asthma experimental protocol.

11. Figure 2. Specify where cytokine levels were measured (BAL, serum, cell culture supernatants, etc.). Although it is stated in a title, first sentence in figure legend is missing this point. 

✔ This modification was done in the revised version

11. Panel d. It looks like OVA treatment induces Treg cells in BAL. Strongly. Now over 24% of CD4+ T cells co-express CD25 and Foxp3. Show a panel with absolute numbers of Treg cells for all experimental conditions. Should you focus on Foxp3+IL-10+ cells in this figure? 

✔ The panel with absolute Treg number was introduced

12. Figure 5, panel a. Incorporate arrows to point on where to look for described results. 

✔ This modification was introduced in the revised version

Comments Reviewer 1:

1. Lines 114-115: Please rephrase. 

This was corrected as suggested by the reviewer. 

2. Lines 118-120: Please rephrase

This was corrected as suggested by the reviewer. 

3. Lines 305-306: 4-6 weeks old mice are not really adult mice. Please adapt that in the whole text.

As stated in the experimental design and in the timeline diagram in Fig 1, mice reached 8-9 weeks old. According to bibliography, 8-12 weeks old in mice physiologically resembles human adulthood (http://dx.doi.org/10.1016/j.lfs.2015.10.025). However, to be more accurate, we introduced the relative ages of mice in the different steps in “experimental design” section, indicating in allergen challenge the starting and ending age of Balb/c mice. To meet the journal recommendations, this protocols has been uploaded to protocols.io (dx.doi.org/10.17504/protocols.io.bbx2ipqe)

4. Line 307: there is a ‘t’ missing in the word ‘first’. 

This was corrected as suggested by the reviewer. 

5. Figure legends: Please indicate the number of experiments and the number of animals used in each experiment.

This was corrected following the reviewer's concern.

6. Figures: Please don’t show bar graphs. Dot blots would be more suitable. And please indicate the significance between specific groups in the graph not only in the legend.

Following this comment, Fig 5 (Fig 6 in the revised manuscript) we changed bar graph for Dot blot spots. Moreover, all graphs of the manuscript were modified and the significance among groups were indicated.

7. Figure 1b: It would increase the readability of graph if the cell types of the different groups would be next to each other. Like this, the difference in eosinophils between PBS/OVA and LPS/OVA is difficult to observe. And please don’t show discontinuous axes.

This was corrected as suggested by the reviewer. The figure is now easier to interpret.

8. Lines 316: It should be Fig 1b.

In the present version of the manuscript, it is Figure 2B

9. Figure 1: It would be interesting to see if there is a difference in cytokines like IL-5, IL-13, IFN-gamma or IL-10.

Regarding this concern, we have already evaluated the levels of IFN-gamma and, they are shown Fig 2A. We have also measured IL-10 in BAL using a commercially available sandwich ELISA kit (#14-7101-67, eBioscience, San Diego, USA) but this cytokine could not be detected. This was probably due to the kit sensitivity (30 pg/ml) or to low concentrations of the cytokine in the BAL. According to the bibliography (doi: 10.1007/978-1-4939-1161-5_5), a more efficient system for cytokine detection is by using a protein transport inhibitor in combination with intracellular staining, with the revealing being performed by immunofluorescence or flow cytometry. In fact, for intracellular IL10 detection, this technique achieved good results as it is shown in the present version of the manuscript (Fig 3F) (dx.doi.org/10.17504/protocols.io.bdxzi7p6). 

The Th2-related cytokines IL-4 and TSLP have also been determined based on the primary role of IL-4 as well as the expression of IL4R in club cells (92-93 line introduction) and on the epithelial involvement of TSLP (78-81 line introduction).

10. Line 332: the term ‘airway’s milieu’ should be specified clearer.

“airway´s milieu” was replaced by “milieu within the airways” and a introduction sentence expresses the reason for the analysis was added – Lines 324-328.

11. Lines 337-341: This part should be explained a bit deeper. Where the cells only stained for IL-10 or IL-10 plus Foxp-3/CD25? Why was the ratio between IL-10 and CD4 cells was calculated?

Referring this comment, cells were stained for Foxp-3/CD25 and IL-10, but following different techniques, flow cytometry and immunofluorescence respectively. By immunofluorescence, the ratio CD4/IL-10was calculated in order to evaluate only IL-10-producing lymphocytes, excluding macrophages and neutrophils. In the revised version, we have introduced a second table with the % of CD25+ cells expressing Foxp-3 by flow cytometry.

12. Line 357-358: The mentioned ‘specific pro-inflammatory’ should be specified more clearly. At the end of the sentence, there is a comma instead of a point. 

In response to this, we suppressed the term and added two new sentences with concepts and clarifications related to the nature of the molecules we are analyzing. Lines 291-293 and 354-356 of Results

13. Figure 3. It would be nice to see the quantification not only for the AB-PAS staining but also for TSLP and pEGFR.

Following this suggestion, we performed a densitometric analysis to quantify TSLP expression by using ImageJ on immunostained sections. A panel graph was also added in Fig 3 (Now Figure 4C) as well as a paragraph in Material and Methods section explaining it. Regarding pEGFR, as its immunolabeling is restricted to the apical cytoplasm of CC after OVA challenge, densitometry would not reflect the actual expression or the changes in pEGFR among the groups.

Attachment Submitted filename: Response to reviewers.docx Click here for additional data file.

# Editor report: Decision Letter 1

**DOI:** 10.1371/journal.pone.0226233.r003

**Authors:** Svetlana P. Chapoval

15 Apr 2020

PONE-D-19-32052R1

Neonatal endotoxin stimulation is associated with a long-term bronchiolar epithelial expression of innate immune and anti-allergic markers that attenuates the allergic response

PLOS ONE

Dear Dr. Noemi Garcia,

Thank you for submitting your manuscript to PLOS ONE. After careful consideration, we feel that it has merit but does not fully meet PLOS ONE’s publication criteria as it currently stands. Therefore, we invite you to submit a revised version of the manuscript that addresses the points raised during the review process.

==============================

ACADEMIC EDITOR:

1. Airway challenge in puberty and adulthood? Is this an appropriate statement for mice?
2. Line 232. Mouse anti-b-actin expression is actually a mouse b-actin detection with anti-b-actin Ab
3. Are website links to the protocol used acceptable for the journal? Like shown in lines 143, 177, 202, 215, 243, 271, and 276?
4. IFNg, PCR,  and many others are standard abbreviations for the journal. No full definition is needed. TSLP ROS, PE, and PerCP probably too. Please correct. Note, that upon their first mentioning in the manuscript text, which is in the abstract, no full definition is provided.

==============================

We would appreciate receiving your revised manuscript by 05/14/2020. When you are ready to submit your revision, log on to https://www.editorialmanager.com/pone/ and select the 'Submissions Needing Revision' folder to locate your manuscript file.

If you would like to make changes to your financial disclosure, please include your updated statement in your cover letter.

To enhance the reproducibility of your results, we recommend that if applicable you deposit your laboratory protocols in protocols.io, where a protocol can be assigned its own identifier (DOI) such that it can be cited independently in the future. For instructions see: http://journals.plos.org/plosone/s/submission-guidelines#loc-laboratory-protocols

Please include the following items when submitting your revised manuscript:

- A rebuttal letter that responds to each point raised by academic editor. This letter should be uploaded as separate file and labeled 'Response to Editor'.
- A marked-up copy of your manuscript that highlights changes made to the original version. This file should be uploaded as separate file and labeled 'Revised Manuscript with Track Changes'.
- An unmarked version of your revised paper without tracked changes. This file should be uploaded as separate file and labeled 'Manuscript'.

Please note while forming your response, if your article is accepted, you may have the opportunity to make the peer review history publicly available. The record will include editor decision letters (with reviews) and your responses to reviewer comments. If eligible, we will contact you to opt in or out.

We look forward to receiving your revised manuscript.

Kind regards,

Svetlana P. Chapoval

Academic Editor

PLOS ONE

Additional Editor Comments (if provided):

Respond to the following comments and revise manuscript accordingly:

1. Airway challenge in puberty and adulthood? Is this an appropriate statement for mice?

2. Line 232. Mouse anti-b-actin expression is actually a mouse b-actin detection with anti-b-actin Ab

3. Are website links to the protocol used acceptable for the journal? Like shown in lines 143, 177, 202, 215, 243, 271, and 276?

4. IFNg, PCR, and many others are standard abbreviations for the journal. No full definition is needed. TSLP ROS, PE, and PerCP probably too. Please correct. Note, that upon their first mentioning in the manuscript text, which is in the abstract, no full definition is provided.

[Note: HTML markup is below. Please do not edit.]

[NOTE: If reviewer comments were submitted as an attachment file, they will be attached to this email and accessible via the submission site. Please log into your account, locate the manuscript record, and check for the action link "View Attachments". If this link does not appear, there are no attachment files to be viewed.]

While revising your submission, please upload your figure files to the Preflight Analysis and Conversion Engine (PACE) digital diagnostic tool, https://pacev2.apexcovantage.com/. PACE helps ensure that figures meet PLOS requirements. To use PACE, you must first register as a user. Registration is free. Then, login and navigate to the UPLOAD tab, where you will find detailed instructions on how to use the tool. If you encounter any issues or have any questions when using PACE, please email us at figures@plos.org. Please note that Supporting Information files do not need this step.

# Author comment: Author response to Decision Letter 1

**DOI:** 10.1371/journal.pone.0226233.r004

20 Apr 2020

Responses to Academic Editor:

1. Airway challenge in puberty and adulthood? Is this an appropriate statement for mice?

Following bibliography and usual terminology, both terms are appropriate for age determination of laboratory mice (Revise in http://dx.doi.org/10.1016/j.lfs.2015.10.025) 

2. Line 232. Mouse anti-b-actin expression is actually a mouse b-actin detection with anti-b-actin Ab

This was corrected as suggested.

3. Are website links to the protocol used acceptable for the journal? Like shown in lines 143, 177, 202, 215, 243, 271, and 276?

During the previous revision process we have already uploaded our laboratory protocols to protocols.io. The website links cited in the Methods section were, actually, the assigned identifier (DOI) provided by protocols.io as submission-guidelines requirements.

Experimental design > http://dx.doi.org/10.17504/protocols.io.bdwwi7fe >141

Immunohistochemical analysis of lung tissue >http://dx.doi.org/10.17504/protocols.io.bdw9i7h6 >175

Immunofluorescence > http://dx.doi.org/10.17504/protocols.io.bdxzi7p6 >199

Flow cytometry >http://dx.doi.org/10.17504/protocols.io.bdx3i7qn >212

Dot Blot Analysis > http://dx.doi.org/10.17504/protocols.io.bbyhipt6 > 241

RNA isolation and gene expression analysis > http://dx.doi.org/10.17504/protocols.io.bdx5i7q6 > 268

Clinical score assessment of the degree of respiratory distress >http://dx.doi.org/10.17504/protocols.io.bbymipu6> 273

4. IFNg, PCR, and many others are standard abbreviations for the journal. No full definition is needed. TSLP ROS, PE, and PerCP probably too. Please correct. Note, that upon their first mentioning in the manuscript text, which is in the abstract, no full definition is provided.

The abbreviations were added to the abstract and we revised the manuscript to keep them a minimum.

Attachment Submitted filename: Response to editor.docx Click here for additional data file.

# Editor report: Decision Letter 2

**DOI:** 10.1371/journal.pone.0226233.r005

**Authors:** Svetlana P. Chapoval

23 Apr 2020

Neonatal endotoxin stimulation is associated with a long-term bronchiolar epithelial expression of innate immune and anti-allergic markers that attenuates the allergic response

PONE-D-19-32052R2

Dear Dr. Luciana Noemi Garcia,

We are pleased to inform you that your manuscript has been judged scientifically suitable for publication and will be formally accepted for publication once it complies with all outstanding technical requirements.

Within one week, you will receive an e-mail containing information on the amendments required prior to publication. When all required modifications have been addressed, you will receive a formal acceptance letter and your manuscript will proceed to our production department and be scheduled for publication.

Shortly after the formal acceptance letter is sent, an invoice for payment will follow. To ensure an efficient production and billing process, please log into Editorial Manager at https://www.editorialmanager.com/pone/, click the "Update My Information" link at the top of the page, and update your user information. If you have any billing related questions, please contact our Author Billing department directly at authorbilling@plos.org.

If your institution or institutions have a press office, please notify them about your upcoming paper to enable them to help maximize its impact. If they will be preparing press materials for this manuscript, you must inform our press team as soon as possible and no later than 48 hours after receiving the formal acceptance. Your manuscript will remain under strict press embargo until 2 pm Eastern Time on the date of publication. For more information, please contact onepress@plos.org.

With kind regards,

Svetlana P. Chapoval

Academic Editor

PLOS ONE

# Editor report: Acceptance letter

**DOI:** 10.1371/journal.pone.0226233.r006

**Authors:** Svetlana P. Chapoval

28 Apr 2020

PONE-D-19-32052R2 

Neonatal endotoxin stimulation is associated with a long-term bronchiolar epithelial expression of innate immune and anti-allergic markers that attenuates the allergic response 

Dear Dr. García:

I am pleased to inform you that your manuscript has been deemed suitable for publication in PLOS ONE. Congratulations! Your manuscript is now with our production department. 

If your institution or institutions have a press office, please notify them about your upcoming paper at this point, to enable them to help maximize its impact. If they will be preparing press materials for this manuscript, please inform our press team within the next 48 hours. Your manuscript will remain under strict press embargo until 2 pm Eastern Time on the date of publication. For more information please contact onepress@plos.org.

For any other questions or concerns, please email plosone@plos.org. 

Thank you for submitting your work to PLOS ONE.

With kind regards,

PLOS ONE Editorial Office Staff

on behalf of

Dr. Svetlana P. Chapoval 

Academic Editor

PLOS ONE
